# Neural stem cells for disease modeling and evaluation of therapeutics for infantile (CLN1/PPT1) and late infantile (CLN2/TPP1) neuronal ceroid lipofuscinoses

**DOI:** 10.1186/s13023-018-0798-2

**Published:** 2018-04-10

**Authors:** Ni Sima, Rong Li, Wei Huang, Miao Xu, Jeanette Beers, Jizhong Zou, Steven Titus, Elizabeth A. Ottinger, Juan J. Marugan, Xing Xie, Wei Zheng

**Affiliations:** 1 National Center for Advancing Translational Sciences, National Institutes of Health, 9800 Medical Center Drive, Bethesda, MD 20892 USA; 2 Department of Gynecologic Oncology, Women’s Reproductive Health Laboratory of Zhejiang Province, Women’s Hospital, School of Medicine, Zhejiang University, Hangzhou, Zhejiang People’s Republic of China; 3 iPSC core, National Heart, Lung and Blood Institute, National Institutes of Health, Bethesda, MD USA

**Keywords:** Neuronal ceroid lipofuscinosis, INCL, LINCL, Lysosomal storage disease, Induced pluripotent stem cells, Neural stem cells, Enzyme replacement therapy, Cyclodextrin, δ-tocopherol

## Abstract

**Background:**

Infantile and late infantile neuronal ceroid lipofuscinoses (NCLs) are lysosomal storage diseases affecting the central nervous system (CNS). The infantile NCL (INCL) is caused by mutations in the *PPT1* gene and late-infantile NCL (LINCL) is due to mutations in the *TPP1* gene. Deficiency in PPT1 or TPP1 enzyme function results in lysosomal accumulation of pathological lipofuscin-like material in the patient cells. There is currently no small-molecular drug treatment for NCLs.

**Results:**

We have generated induced pluripotent stem cells (iPSC) from three patient dermal fibroblast lines and further differentiated them into neural stem cells (NSCs). Using these new disease models, we evaluated the effect of δ-tocopherol (DT) and hydroxypropyl-β-cyclodextrin (HPBCD) with the enzyme replacement therapy as the control. Treatment with the relevant recombinant enzyme or DT significantly ameliorated the lipid accumulation and lysosomal enlargement in the disease cells. A combination therapy of δ-tocopherol and HPBCD further improved the effect compared to that of either drug used as a single therapy.

**Conclusion:**

The results demonstrate that these patient iPSC derived NCL NSCs are valid cell- based disease models with characteristic disease phenotypes that can be used for study of disease pathophysiology and drug development.

**Electronic supplementary material:**

The online version of this article (10.1186/s13023-018-0798-2) contains supplementary material, which is available to authorized users.

## Background

The neuronal ceroid lipofuscinoses (NCLs) are a group of neurodegenerative diseases with symptoms including progressive vision loss culminating in blindness, cognitive and motor decline, and seizures [1]. There are multiple subgroups of NCL disease based on the onset and severity of the disease. Infantile neuronal ceroid lipofucinosis (INCL, also called CLN1) is caused by mutations in the *PPT1* gene that encodes the enzyme Palmitoyl-Protein Thioesterase 1 (PPT1). Patients with INCL generally develop symptoms around 18 months of age including visual defects and blindness, motor and cognitive deficits; seizures and death occur ultimately at 8 to 13 years of age [2, 3]. Late infantile NCL (LINCL, also called CLN2) results from mutations in the *TPP1* gene that encodes the enzyme Tripeptidyl Peptidase-1 (TPP1). Symptoms in patients with LINCL usually appear between 2 and 4 years of age; death occurs between 8 and 12 years of age [3]. The typical early signs are loss of muscle coordination (ataxia) and seizures, along with progressive mental deterioration. Neurological deterioration and the accompanying brain atrophy ultimately leads to death [4].

Deficiency of lysosomal enzymes PPT1 in CLN1 or TPP1 in CLN2 results in lysosomal accumulation of lipids and subsequently the enlargement of lysosomes in patient cells [5, 6]. Enzyme replacement therapy (ERT) is currently available to treat several lysosomal storage diseases including Gaucher, Fabry, Pompe, Mucopolysaccharidosis (MPS) types I, MPS-II and MPS-VI [7–9]. ERT is suitable for the peripheral symptoms (kidney, liver, heart, lung and spleen) but not for the neuronal symptoms because the recombinant enzyme cannot penetrate the blood-brain-barrier [10, 11]. In late April of 2017, FDA approved Brineura (Cerliponase alfa) for the treatment of CLN2, also known as TPP1 deficiency. However, there is no small-molecule drug treatment for both CLN1 and CLN2 [12]. Other therapies such as gene therapy are still under development [11].

In our previous research, δ-tocopherol reduced the lysosomal cholesterol accumulation in patient cells of Niemann Pick disease type C [13]. The mechanism of action for δ-tocopherol has been linked to the increase in lysosomal exocytosis in the patient cells. It also reduced the enlarged lysosome size in Niemann-Pick type A (NPA) patient fibroblasts (FIB) [14]. Another compound, hydroxypropyl-β-cyclodextrin (HPBCD) had been reported to reduce lysosomal cholesterol accumulation which is more potent in patient neural stem cells (NSCs), differentiated from induced pluripotent stem cells (iPSCs), than in patient fibroblasts [15]. HPBCD also reduced sphingomyelin accumulation and enlarged lysosomes in NPA neural stem cells [14]. Based on these findings, we examined the effects of δ-tocopherol and HPBCD in a new, more relevant, cell-based INCL and LINCL disease models.

To establish the neurological disease model for evaluating the efficacy of the drugs, we carried out the reprogramming of patient cells to induced pluripotent stem cells (iPSCs). Here we report the generation of patient iPS cell lines from one CLN1 (INCL) and two CLN2 (LINCL) patient fibroblast lines. These patient iPSCs were further differentiated into NSCs that exhibited the characteristic disease phenotype of reduced PPT1 or TTP1 protein level and enlarged lysosomes. Using these NCL NSCs, we evaluated the pharmacological effects of ERT, δ-tocopherol, and HPBCD. Our results demonstrate that the neural stem cells differentiated from NCL iPSCs are useful disease models for further study of NCL pathophysiology and for drug development to find treatments for NCLs.

## Methods

### Materials

Hoechst 33,342 (catalog number H3570), CELLstart (A1014201) and lysoTracker red (L7528) were obtained from Thermo Fisher Scientific (MA, USA). Filipin (9765) was ordered from Sigma-Aldrich (MO, USA). Nuclear Red DCS1 was obtained from AAT Bioquest (17,552, CA). Once purchased from Sigma-Aldrich (MO, USA) δ-tocopherol was purified by HPLC to a purity greater than 99%. Black, clear bottom, tissue-culture treated 96-well plates (655090) were purchased from Greiner Bio-One (Monroe, NC). Matrigel (354277) was obtained from Corning (New York, USA). HPBCD was obtained from Roquette America (IL, USA). PSC Neural Induction Medium (A1647801) was purchased from Thermo Fisher Scientific; it contained Neurobasal medium and 1X Neural Induction Supplement. Lamp1 (ab25630) was purchased from Abcam (Cambridge, MA). The secondary antibodies, donkey anti-rabbit IgG labeled with Alexa Fluor 594 (A-21207) and donkey anti-mouse IgG labeled with Alexa Fluor 488 (A-21202), were obtained from Thermo Fisher Scientific.

### Generation of iPS cell lines

Primary human dermal fibroblast cell lines were purchased from the Coriell Cell Repository (Camden, NJ, USA), including wide type (GM05659): one male CLN1 patient (GM20389), as only one CLN1 patient fibroblast cell line was available in Coriell Cell Repository; one female CLN2 patient (GM16485) and one male CLN2 patient (GM16486). The GM20389 line carries mutations in the *PPT1* gene (a T-to-C transition at nucleotide 739 in exon 8 and a G-to-A transition at nucleotide 3 in exon 1). The GM16485 and GM16486 lines carry mutations in the *TPP1* gene. The former (GM16485) contains a C-to-T transition at nucleotide 379 in exon 4 and has a C-to-T transition at nucleotide 622 in exon 6. The later (GM16486) has a G-to-A transition at nucleotide 380 in exon 4 and has a G to C transversion in intron 5. The primary fibroblasts were cultured in DMEM medium with 10% fetal bovine serum (FBS). The cells were reprogrammed into induced pluripotent stem cells using the non-integrating CytoTune–Sendai viral vector kit (A16517, Thermo Fisher Scientific) following the method described previously [16]. Briefly, fibroblasts were plated at a high density in a 48-well plate and the CytoTune-iPS 2.0 Sendai reprogramming kit was used to infect cells according to instructions. At day 4, cells were re-plated onto a Matrigel coated dish in E8-media based reprogramming media, and fed every other day until day 20 and individual colonies were passaged by the ethylenediaminetetraacetic acid (EDTA) dissociation method into separate wells in E8 medium. The selected iPSC colonies (two for each patient sample) were further cultured beyond 15 passages.

### Fluorescence-activated cell sorting (FACS) analysis and karyotyping of iPS cells

The iPS cells were harvested from 6-well plates using TrypLE Express enzyme (12,605,010, Thermo Fisher Scientific). Cells were fixed with 4% paraformaldehyde for 10 min at room temperature and then washed with phosphate buffered saline (PBS). Prior to FACS analysis, cells were permeabilized with 0.2% Tween-20 in PBS for 10 min at room temperature and stained with fluorescein isothyocyanate (FITC) conjugated anti-Tra-1-60 (FCMAB115F) and Alexa Fluor 488 conjugated anti-Nanog (FCABS352A4) obtained from Merck Millipore with the concentrations recommended in the instruction. Non-immune controls were used at 0.5 μl per 50 μl reaction with mouse-IgG2b-FITC (MABC006F, Merck Millipore) and rabbit IgG isotype-AlexaFluor 488 conjugate (4340S, Cell Signaling Technology). Cells were then analyzed using a C6 Flow Cytometer System (653,118, BD Biosciences).

The iPS cells were seeded in T-25 flasks. The G-banding karyotype analysis was conducted at WiCell Research Institute (Madison, WI, USA). Cell harvest, slide preparation and G-banded karyotyping were performed using standard cytogenetic protocols. Cells were incubated with ethidium bromide and colcemid and then placed in hypotonic solution followed by fixation. Metaphase cell preparations were stained with Leishman’s stain. A total of 20 randomly selected metaphases were analyzed by G-banding for each cell line.

### NSCs induction from iPSCs and immunofluorescence staining of NSC protein markers

The resulting iPSCs were differentiated to NSCs using the PSC Neural Induction Medium (A1647801, Thermo Fisher Scientific) following the protocol from the manufacture. Briefly, iPSCs were cultured in feeder-free Essential 8 Medium (A1517001, Thermo Fisher Scientific) on 6-well plates coated with Matrigel hESC-qualified matrix (354,277, Corning). When cells reached 70 to 80% confluency, they were dissociated with 0.5 mM EDTA buffer. Next the cells were reseed onto the Matrigel pre-coated 6-well plates at 3 × 10^5^ cells/well in the E8 medium with 10 μM Rho-associated coiled-coil kinase (ROCK) inhibitor Y-27632 (1254, Tocris Bioscience). Cell culture medium was then changed to the complete PSC Neural Induction Medium after cell attachment. The cells were cultured for another 7 days with medium changes every other day. At day 7 of neural induction, the initial NSCs were dissociated with the StemPro cell dissociation reagent (A11105) and plated in the Matrigel pre-coated T75 flasks for further expansion in the Neural Expansion Medium (Neurobasal medium, Advanced DMEM/F12, A12634 and 1X Neural Induction Supplement).

For immunofluorescence staining, NSCs were fixed in 4% paraformaldehyde for 15 min, rinsed with PBS, and permeabilized with 0.3% Triton X-100 for 15 min, followed by incubation individually with four primary antibodies including Oct4 (1:50 dilution), Sox1 (1:50 dilution), Sox2 (1:50 dilution), and Nestin (1:50 dilution) using the human neural stem cell immunocytochemistry kit (A24354, Thermo Fisher Scientific) for overnight at 4 °C. After washing with PBS, a corresponding secondary antibody conjugated with Alexa Fluor 647 (1:250 dilution) was added. Cells were then stained with Hoechst 33,342 for 20 min after a wash and imaged using an IN Cell Analyzer 2200 imaging system (GE Healthcare) with 20X objective lens and Cy5, FITC and DAPI filter sets.

### LysoTracker-red dye, Nile red, and Filipin staining experiments

LysoTracker dye stains acidic compartments in cells, visualizing enlarged lysosomes in patient cells at the proper dye concentration compared to wild type (WT) cells. Briefly, fibroblasts (1500/well) or NSCs (1 to 2 × 10^4^/well) were seeded in black, clear-bottom, 96-well plates and treated with compounds for 3 days. The cells were washed with PBS and incubated with 100 μl/well 50 nM LysoTracker red dye at 37 °C for 1 h. The cells were fixed in 100 μl/well 3.2% paraformaldehyde solution containing 1 μg/ml Hoechst 33,342 in PBS and incubated at room temperature for 30 min. After cell wash, the cells were imaged in the IN Cell Analyzer 2200 imaging system using DAPI and DsRed filter sets.

Nile red dye stains accumulated lipids and lipid droplets in cells. It has been reported that the yellow-gold fluorescence (excitation, 450–500 nm; emission, > 528 nm) detects cytoplasmic lipid droplets better than red fluorescence (excitation, 515–560 nm; emission, > 590 nm) [17]. In this experiment, the Nile red dye staining was used to evaluate the accumulation of lipids in NCL patient cells. Briefly, fibroblasts cells (1500/well) or NSCs (3000–5000 cells/well) were seeded in black, clear-bottom, 96-well plates for 3–4 days in the presence or absence of compound treatment. The cells were washed with warm medium, to which was added 100ul/well Nile red dye solution in medium (1:1000 dilution, 1 μM final concentration) and incubated at 37 °C for 10 min. Next, the cells were fixed in 100 μl/well 3.2% paraformaldehyde solution containing 1 μg/ml Hoechst 33,342 in PBS and incubated at room temperature for 30 min. After cell wash, the cells were imaged in the IN Cell Analyzer 2200 imaging system using 40X objective lens with the filter sets of Texas Red (TR)/TR (excitation, 575 ± 25 nm; emission, 620 ± 30 nm) for deep red fluorescence, FITC/YFP (excitation, 475 ± 28 nm; emission, 548 ± 22 nm) for yellow-gold fluorescence and DAPI/DAPI (excitation, 390 ± 18 nm; emission, 432 ± 48 nm) for nuclei staining.

Filipin dye binds specifically to unesterified cholesterols in cells which produces blue fluorescence. Fibroblasts cells (1500/well) or NSCs (20,000/well) were seeded in 96-well plates for four days in the presence or absence of compound treatment. After washing cells with PBS, cells were fixed in 200 μl/well of 3.2% paraformaldehyde in PBS and incubated at room temperature for 30 min. The cells were then stained with 50 μg/ml Filipin for 1 h at room temperature, followed by nuclei staining with the nuclear-red dye (8 μM final) for 15 min in the dark. After the cells were washed with PBS twice, 100 μl/well PBS was added and the plates were imaged in the IN Cell Analyzer 2200 imaging system using nuclear-red and FITC filter sets.

Images were analyzed with the multi-target analysis protocol (IN Cell Imaging analysis software, GE Healthcare). Nuclei were segmented using the top-hat segmentation method with a minimum area set at 75 μm^2^ and a sensitivity set at 50. Stained lysosomes were identified as “organelles” and were segmented using the multiscale top-hat algorithm. Settings for lysosome detection included identification of granules ranging in size from 0.5 to 45 μm and a sensitivity setting of 60. Total organelle intensity was calculated by a user-defined threshold for organelle intensity.

### ERT with recombinant human palmitoyl-protein thioesterase 1 (PPT1) and tripeptidyl-peptidase 1 (TPP1)

Human recombinant PPT1 (14703-H08H) was purchased from Sino Biological (Beijing, China), and TPP1 (2237-SE-010) was ordered from R&D Systems (Minneapolis, MN). The PPT1 enzyme was prepared and stored in sterile water at a concentration of 0.25 mg/ml, and TPP1was supplied at a concentration of 0.44 mg/ml in 25 mM Tris and 150 nM NaCl, pH 7.5. The NSCs were seeded in 96-well plates pre-coated with the CELLstart matrix (1:50 dilution into Dulbecco’s phosphate buffered saline (DPBS) with Ca^2+^ and Mg^2+^ at 37 °C and incubated for 1 h). On the second day, PPT1 (final concentration 0.2 μM) was added to *PPT1*^*E8/E1*^ NSCs, and TPP1 (final concentration 0.2 μM) to *TPP1*^*E4/E6*^ and *TPP1*^*E4/IVS5*^ NSCs and incubated at 37 °C for 4 h. The medium was changed to remove the recombinant enzymes in the solution and the cells were cultured in 10% serum containing NSC expression medium. After incubation for 72 h, the NSCs were stained using the LysoTracker red dye or harvested for western blotting.

### Immunofluorescence staining for subunit c and LAMP-1

NSC cells were seeded in 96 well matrigel pre-coated plates at a density of 12,000 to 20,000 cells/well in the Neural Expansion Medium and incubated at 37 °C, 5% CO_2_ for one day. The cells were fixed with 4% paraformaldehyde and immunostaining was carried out with the anti-adenosine triphosphate (ATP) synthase subunit c antibody (Abcam, Catalog No. ab181243) and anti-LAMP-1 antibody (Abcam, Catalog No. ab25630) as described above. After washing the cells, they were imaged in the IN Cell Analyzer 2200 imaging system using FITC, DsRed and DAPI filter sets.

### Western blot

Western blots were performed as described previously [18].Cells were harvested and resuspended in lysis buffer for protein extraction. Total protein, ranging from 25 to 50 μg for each sample, was subjected to electrophoresis using NuPAGE™ 4–12% Bis-Tris Protein Gel (Thermo Fisher Scientific). Primary antibodies against the proteins of PPT1 (ab89022), TPP1 (ab54685) were purchased from Abcam. The control β-actin antibody (4970S) was obtained from Cell Signaling Technology. After applying the secondary antibody, Luminata Forte Western horseradish peroxidase (HRP) substrate (WBLUF0500, MilliporeSigma) was used for visualization. Cumulative gray level of Western blot bands was analyzed for quantitation using the UVP Software (Ultra-Violet Products Ltd., CA).

### ATP content assay for cell viability

An ATP content assay kit (ATPlite, PerkinElmer) was used to measure cell viability to monitor compound cytotoxicity. Cells were seeded at 3000–5000 cells/well in 100 μl medium in white, solid 96-well plates and incubated for 24 h. Cells were cultured and treated with compounds as described above. After 3 days of incubation, 100 μl/well of ATP content reagent mixture (prepared according to the manufacture’s instruction) was added to the assay plates followed by incubation at room temperature for 10–15 min. The luminescence signal was determined in the luminescence mode of the ViewLux Plate reader (PerkinElmer).

### Lysosomal pH measurement

The lysosomal PH was determined using a fluorescence-labeled dextran dye (pHrodo™ dextran, Thermo Fisher) following the method established previously [13, 19]. This dye emits strong fluorescence signal in an acidic environment while exhibiting minimal fluorescence signal at neutral pH. Briefly, NCL patient NSCs (1–2 × 10^4^ cells/well) were seeded in black, clear-bottom, 96-well plates and treated with compounds for 3 days. The cells were washed with NSC culture medium and incubated with NSC cultural medium containing 1 μg/ml Hoechst 33,342 at 37 °C for 30 min. Then the cells were stained with 100 μl/well 20μg/ml pHrodo™ dextran dye at 37 °C for 2 h. After washed twice with Live Cell Imaging Solution (Thermo Fisher Scientific), the cells were imaged in the IN Cell Analyzer 2200 imaging system using Cy3 and DAPI filter sets.

### Data analysis and statistics

Concentration-response curves were analyzed and IC_50_ values were calculated using the Prism 5 software (GraphPad Software, CA, USA). Results in the figures were expressed as mean of triplicates ± standard error of the mean (SEM). Unless otherwise stated, unpaired t-tests were used to determine significance (* *P* < 0.05 and ** *P* < 0.01).

## Results

### Generation of iPS cell lines from patient fibroblasts and NSC differentiation

A Sendai viral vector kit was used to reprogram the patient iPS dermal fibroblast lines including one INCL (GM20389) and two LINCL (GM16485 and GM16486) from Coriell Cell Repository (Additional file 1: Figure S1A). Two iPSC colonies were established for each of the patient lines as HT146B/HT146F from GM20389, HT140A/140E from GM16485, and HT264A/264B from GM16486 (Table 1). These iPS cell lines were passaged over 15 times and no abnormality was observed in morphology and growth rate. Normal karyotypes were also observed in these iPS cell lines (Additional file 1: Figure S1B). Flow cytometric analysis showed that these iPS cells expressed the pluripotency markers Nanog and Tra-1-60 (Additional file 1: Figure S2). The pluripotent state of these cells was confirmed by positive staining of pluripotent markers including SOX2, SSEA4, TRA-1-60, Nanog and Oct4 (Additional file 1: Figure S3). The iPSCs were then differentiated to NSCs using the Neural Induction Medium kit. The NSC markers including Nestin, PAX6, Sox1 and Sox2 were positively stained in these neural stem cell lines derived from patient iPSCs (Additional file 1: Figure S4A, B). Together, the results demonstrated establishment of three iPSC lines from INCL and LINCL patient cells; these iPSCs were successfully differentiated into NSCs.

**Table 1:** Summary of human cell lines used in the study

Subject	Fibroblasts	iPSC lines	Sex	Genotype/Phenotype	Genotype designation
WT	GM05659	HT268A	Male	Wild Type	WT
NPC1	GM03123	HT237A	Female	NPC1 c.709C > T (p.Pro237Ser) and c.3182 T > C (p.Ile1061Thr)	NPC1
CLN1	GM20389	HT146B, F	Male	PPT1 c.739 T > C (p.Tyr247His) and c.3G > A (p.Met1Ile)	*PPT1* ^*E8/E1*^
CLN2	GM16485	HT140A, E	Female	TPP1 c.379C > T (p.Arg127Ter) and c.622C > T, (p.Arg208Ter)	*TPP1* ^*E4/E6*^
CLN2	GM16486	HT264A, B	Male	TPP1 c.380G > A (p.Arg127Gln) and IVS5-1G > C	*TPP1* ^*E4/IVS5*^

### Lipid accumulation and enlarged lysosomes in NCL fibroblasts and NSCs

**Fig. 1:**
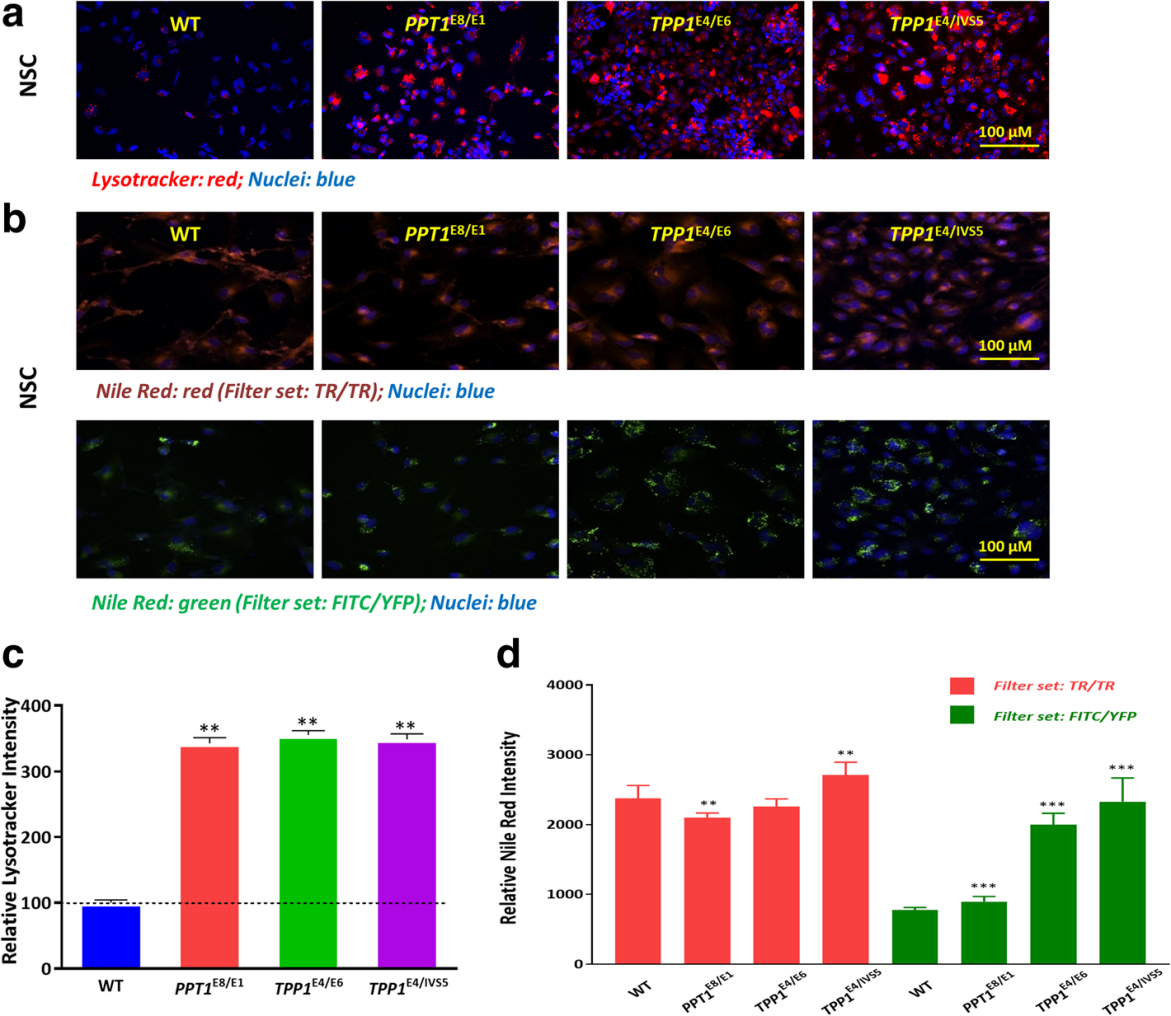
Enlarged lysosomes and lipid accumulation in NCL patient NSCs. Increased LysoTracker dye staining (**a**) indicates enlarged lysosomes and a strong Nile red staining (**b**) indicates cytoplasmic lipid droplet accumulation in NCL NSCs compared to the WT control cells. Representative image of LysoTracker dye and Nile red staining were shown with 40X objective lens. Quantification of the acidic compartment (**c**) revealed significantly enlarged lysosomes in NCL NSCs and quantification of the cytoplasmic lipid droplet accumulation (**d**) was found significantly increased in NCL NSCs. Data are displayed as mean ± SD. ** *P* < 0.01 vs. WT control

To determine whether the lysosomes were enlarged in the patient cells due to the accumulation of lipids, LysoTracker dye staining was performed to visualize enlarged lysosomes. We found elevated LysoTacker staining in both parental NCL patient fibroblasts (Additional file 1: Figure S5A and C) and NSCs (Fig. 1a and c). The LysoTracker dye staining increased 3.8, 2.5 and 2.8-fold in the patient fibroblast lines (*PPT1*^E8/E1^, *TPP1*^E4/E6^ and *TPP1*^E4/IVS5^, respectively) (Additional file 1: Figure S5C) compared to that in the WT control. Similarly, the LysoTracker dye staining increased 3.4, 3.5 and 3.4-fold in the *PPT1*^E8/E1^, *TPP1*^E4/E6^ and *TPP1*^E4/IVS5^ NCL NSC lines, respectively, compared to that in the WT control (Fig. 1c).

To determine the lipid accumulation in patient cells, we performed the Nile red dye staining experiment. We found that the Nile red dye staining in patient NCL fibroblasts was significantly higher than that in the wild type cells (Additional file 1: Figure S5B and D). In NCL patient NSCs, only slight increase of Nile red-staining was observed compared to the WT control in the deep red fluorescence channel, while the fluorescence intensity in the yellow-gold channel was greatly increased. The yellow-gold fluorescence increased 1.2, 2.6 and 3.0-fold in the *PPT1*^E8/E1^, *TPP1*^E4/E6^ and *TPP1*^E4/IVS5^ NCL NSC lines, respectively, compared to that in the WT control (see Fig. 1b and d).

Filipin dye stains unesterified cholesterols that accumulated in the lysosomes of many patient cells with lysosomal storage diseases, especially the Niemann Pick disease type C. But the Filipin staining was negative in both paired patient fibroblasts and NSCs (Additional file 1: Figure S6), indicating an absence of lysosomal accumulation of unesterified cholesterols in these NCL patient cells.

Together, these results indicate a significant enlargement of lysosomes and abnormal accumulation of cytoplasmic lipid droplets in all patient NSCs, similar to those in parental fibroblast lines. There is no lysosomal accumulation of unesterified cholesterols in either NCL fibroblasts or NSCs. The lysosomal enlargement and cytoplasmic lipid droplets accumulation in the patient NSCs may serve as a disease phenotype for the cell-based disease models.

### ERT with recombinant PPT1 or TPP1 reduced the enlarged lysosomes in NCL NSCs and fibroblasts

**Fig. 2:**
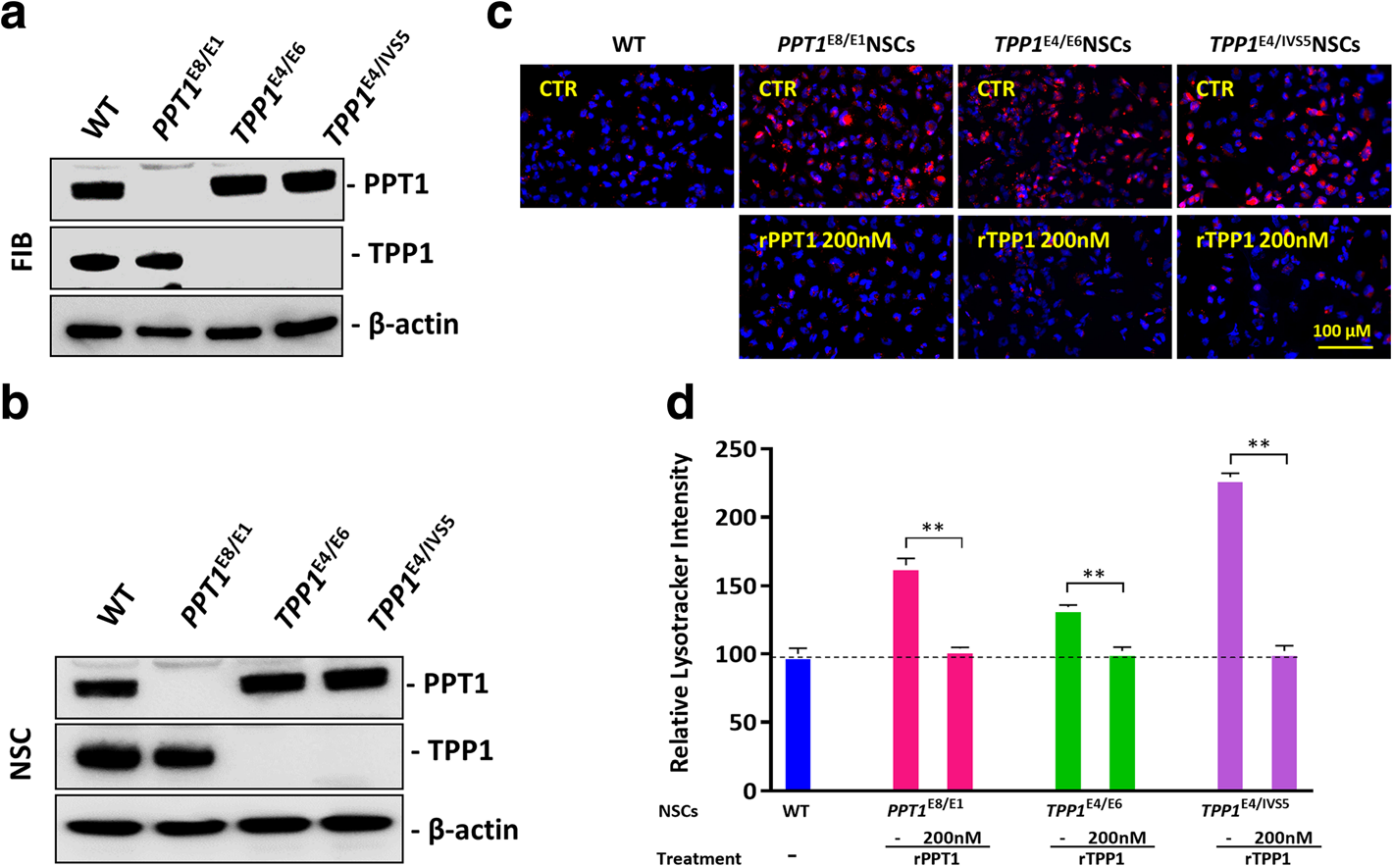
Effect of recombinant human PPT1/TPP1 protein on reducing enlarged lysosomes in NCL patient NSCs. The western blot analysis (**a**, **b**) showed that there is a PPT1 deficiency in *PPT1*^E8/E1^ fibroblasts and NSCs, and also there is no TPP1 expression detected in *TPP1*^E4/E6^ and *TPP1*^E4/IVS5^ fibroblast and NSCs. The treatment of NCL NSCs with 200 nM rPPT1/rTPP1 significantly reduced the LysoTracker dye staining(**c**), with an effect nearly 99.9% in the NCL NSC lines treated with ERT (**d**). The images were taken with 40X objective lens. Data are displayed as mean ± SD. ** *P* < 0.01

Significant deficiency in the mutant enzymes, PPT1 in INCL and TPP1 in the LINCL, was confirmed in patient fibroblasts and NSCs by Western blot analysis (Fig. 2a, b). Almost no residual PPT1 was left in the patient *PPT1*^*E8/E1*^ cells and no TPP1 was present in the *TPP1*^*E4/E6*^ NSCs or *TPP1*^*E4/IVS5*^ NCSs.

The effect of enzyme replacement therapy (ERT) with recombinant WT PPT1 (rPPT) or recombinant TPP1 (rTPP1) was evaluated in the patient NSCs. Treatment with 200 nM of recombinant PPT1 in the *PPT1*^*E8/E1*^ NSCs or rTPP1 in the *TPP1*^*E4/E6*^ NSCs or *TPP1*^*E4/IVS5*^ NSCs significantly reduced LysoTracker dye staining in these patient cells (Fig. 2c). ERT reduced the pathological increase of LysoTracker dye staining in all the NSCs to almost normal levels when compared to WT (Fig. 2d). The results indicated that the ERT with rPPT1 or rTPP1 proteins rescued the disease phenotype in NSCs of enlarged lysosomes due to the lipid accumulation, similarly to that in other lysosomal storage disease cells [14].

### Reduction of enlarged lysosomes in NCL NSCs by δ-tocopherol

**Fig. 3:**
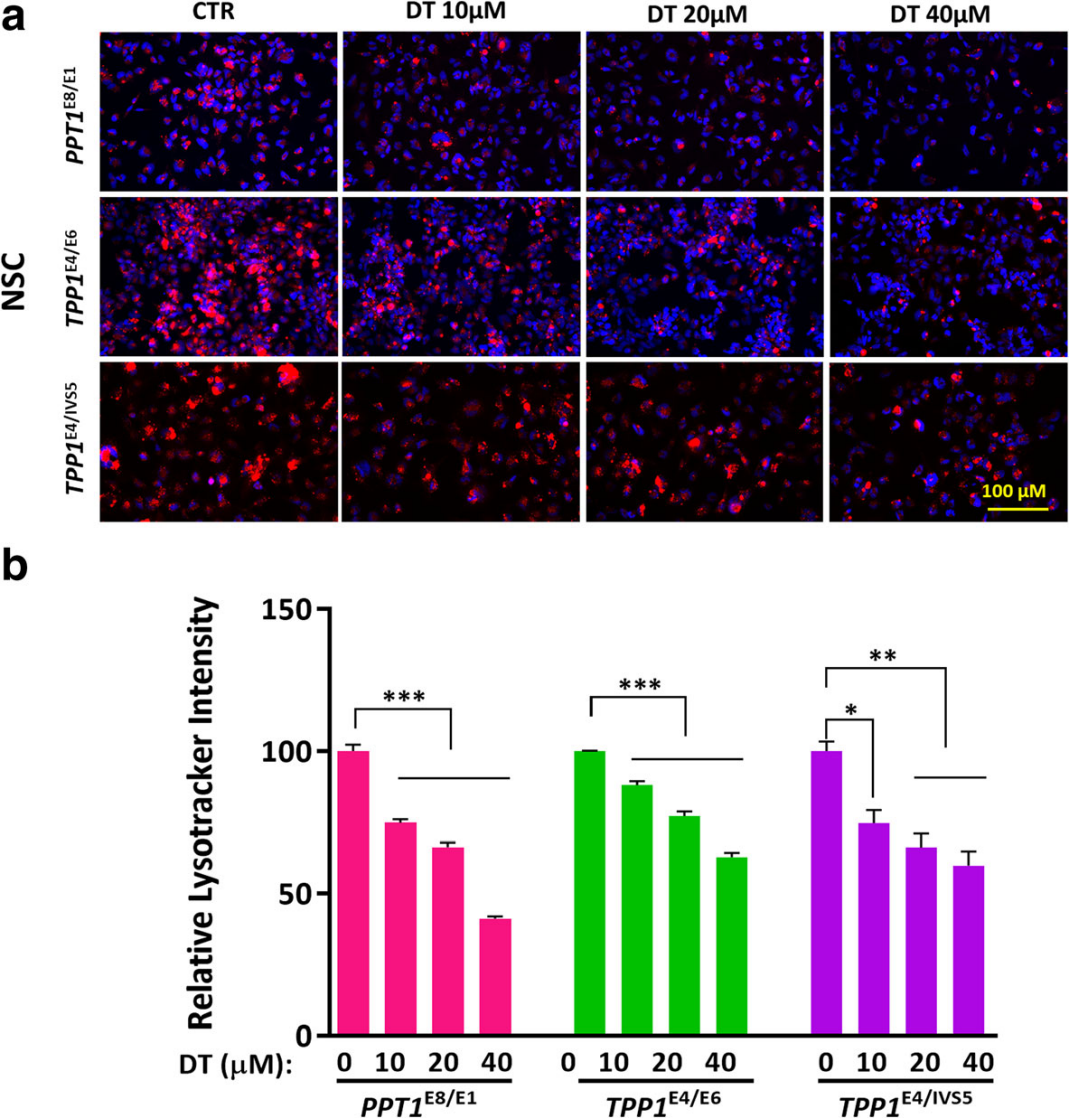
Effect of DT on reducing enlarged lysosomes in NCL patient NSCs. DT dose-dependently reduced the LysoTracker staining (**a**) in NCL NSCs. The quantitative analysis of LysoTracker fluorescence (**b**) revealed that clearance of enlarged lysosomes ranged from 22.8% in the *TPP1*^*E4/E6*^ NSCs to 33.8% in the *PPT1*^*E8/E1*^ NSCs after the treatment with 20 μM DT. The images were taken with 40X objective lens. Data are displayed as mean ± SD. * *P* < 0.05, ** *P* < 0.01, *** *P* < 0.001

Significant reduction of lysosomal cholesterol accumulation and decreased enlarged lysosomes in the Niemann Pick disease cells is achieved by δ-tocopherol [15]. The mechanism of action of δ-tocopherol has been linked to an increase in lysosomal exocytosis of excessive storage materials in the disease cells [13]. The effect of δ-tocopherol (DT) on enlarged lysosomes in the NCL NSCs was evaluated. We found that δ-tocopherol significantly reduced the LysoTracker dye staining in the patient NSCs (Fig. 3a). The IC_50_ values were 11.8 μM in the *PPT1*^*E8/E1*^ NSCs, and 21.3 μM in the *TPP1*^*E4/E6*^ NSCs and 15.5 μM in the *TPP1*^*E4/IVS5*^ NSCs. The reduction effect on LysoTracker staining with the treatment of 20 μM DT ranged from 22.8% in the *TPP1*^*E4/E6*^ NSCs to 33.8% in the *PPT1*^*E4IVS5*^ NSCs (Fig. 3b). 40 μM DT treatment showed stronger effects on reduction of lysoTracker staining compared to 20 μM DT, but it also exhibited cytotoxic effects on those NSCs based on the results from the cytotoxicity assay (Additional file 1: Figure S7). The results indicated that DT significantly reduced enlarged lysosomes in the NCL NSCs.

### Hydroxypropyl-β-cyclodextrin decreased enlarged lysosomes in NCL NSCs

**Fig. 4:**
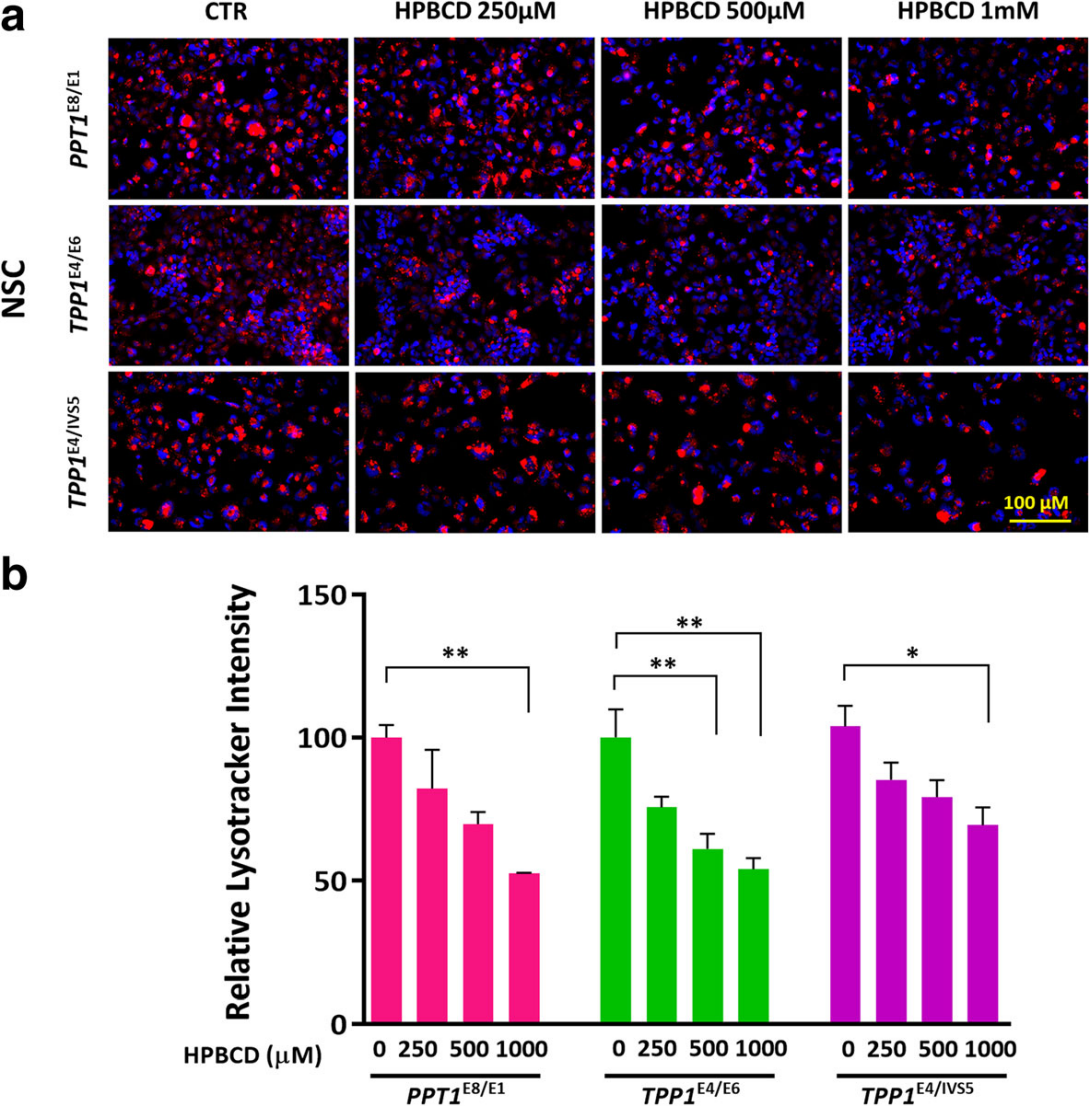
Hydroxypropyl-β-cyclodextrin (HPBCD) ameliorated enlarged lysosomes in NCL NSCs. **a** Representative image of HPBCD’s effect on reducing LysoTracker staining in NCL NSCs. The maximum reduction of enlarged lysosomes ranged from 31% in *TPP1*^*E4/IVS5*^ NSCs to 47% in *PPT1*^*E8/E1*^ NSCs after 1 mM HPBCD treatment (**b**). The images were taken with 40X objective lens. Data are displayed as mean ± SD. * *P* < 0.05, ** *P* < 0.01, *** *P* < 0.001

HPBCD reportedly decreased cholesterol accumulation and reduced enlarged lysosomes in patient cells of Niemann Pick disease type C1 [15, 20]. The effect of HPBCD on enlarged lysosomes in the NCL NSCs was examined using the LysoTracker dye staining assay (Fig. 4a). We found that 1 mM HPBCD significantly reduced the increased LysoTracker dye staining in patient NSCs, ranging from 31% in *TPP1*^*E4/IVS5*^ NSCs to 47% in *PPT1*^*E8/E1*^ NSCs (Fig. 4b). The results indicated HPBCD reduces enlarged lysosomes in the NCL NSCs although high drug concentration was needed.

**Fig. 5:**
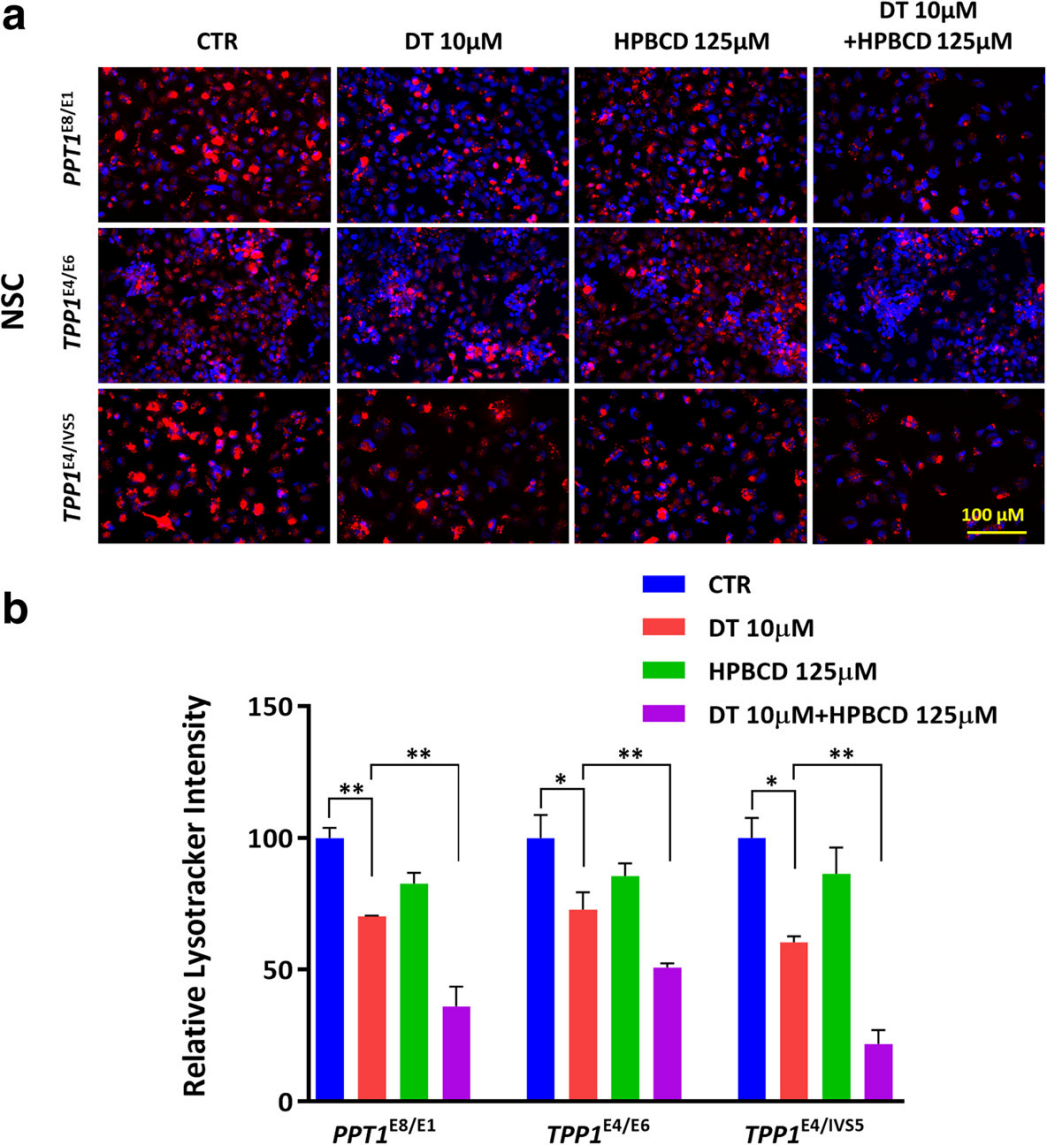
The synergistic effect of HPBCD with DT on reduction of enlarged lysosomes in NCL NSCs. **a** Representative image of a combination of HPBCD and DT on reduction of LysoTracker staining in NCL NSCs compared to DT treatment. **b** The reduction of enlarged lysosomes ranging from 49% in *TPP1*^*E4/E6*^ NSCs to 78% in *TPP1*^E4/IVS5^ NSCs. The images were taken with 40X objective lens. Data are displayed as mean ± SD. * *P* < 0.05, ** *P* < 0.01

Since the concentration of HPBCD required to reduce enlarged lysosomes is high, we next examined a combination of HPBCD with δ-tocopherol in NCL NSCs. Treatment with a combination of 125 μM HPBCD and 10 μM δ-tocopherol improved the potencies of both compounds to reduce enlarged lysosomes in the patient cells, compared with that of HPBCD or δ-tocopherol alone (Fig. 5a), with the effect ranging from 51% in *TPP1*^*E4/E6*^ NSCs to 22% in *TPP1*^E4/IVS5^ NSCs (Fig. 5b). The two-compound combination effective reduced the concentrations of both compounds needed for reduction of lipid accumulation in the patient cells, indicating a synergistic effect of the combination of HPBCD with δ-tocopherol.

To further eliminate the potential effects of DT and HPBCD on the pH of late endosomes and lysosomes, we treated the NCL NSCs with fluorescent dextran dye (pHrodo™ dextran). Similarly to that previously reported [13], we found that the treatment with DT and HPBCD did not alter the fluorescence intensity of this dye in the acidic vesicles in NCL patient cells (Additional file 1: Figure S8A and B), indicating both compounds did not change the lysosomal pH.

### Effects of DT on cytoplasmic lipid droplets accumulation in NCL NSCs

**Fig. 6:**
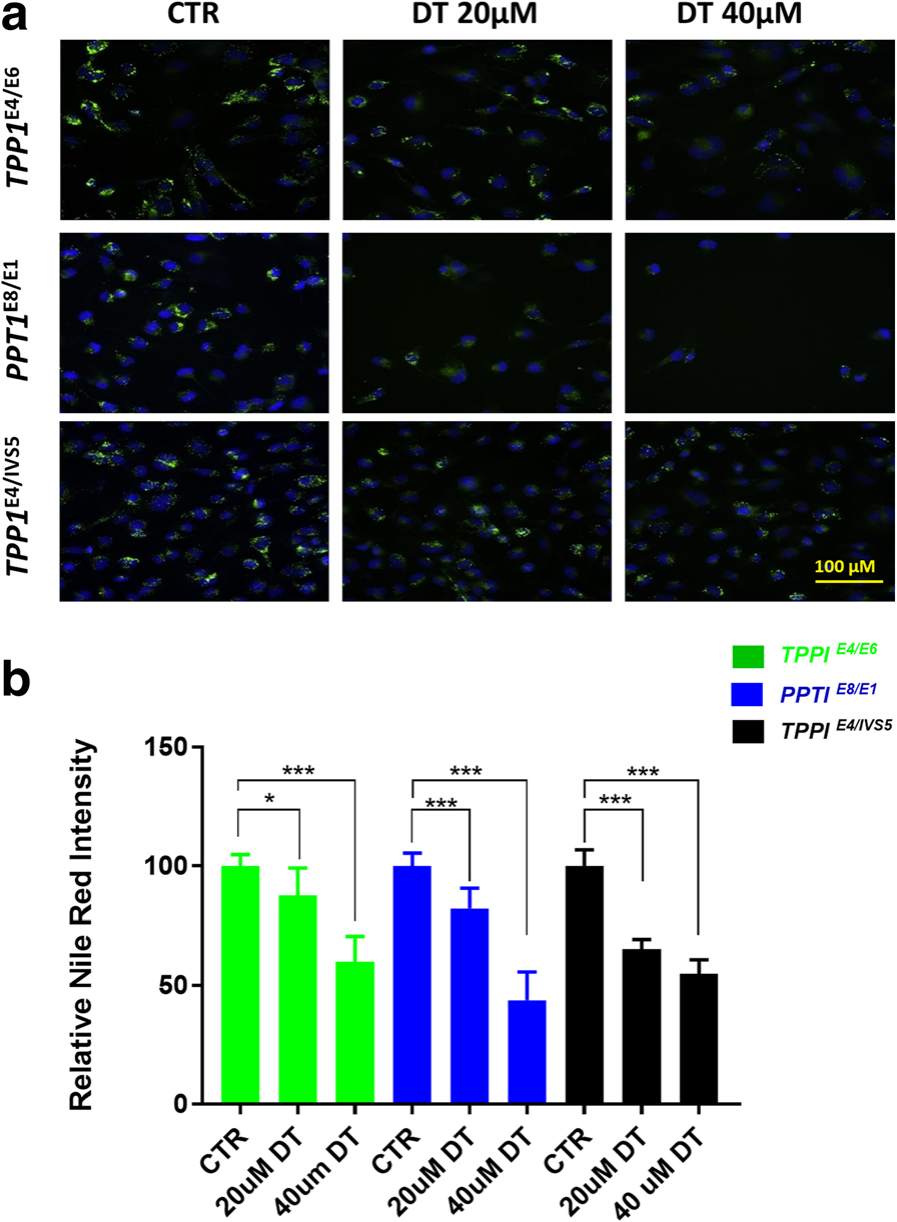
Effect of DT on cytoplasmic lipid droplets accumulation in NCL patient NSCs. DT dose-dependently reduced the Nile red staining (**a**) in NCL NSCs. The quantitative analysis of Nile red fluorescence (**b**) revealed that clearance effect of lipid droplets ranged from 12.4% in the *TPP1*^*E4/E6*^ NSCs to 34.9% in the *TPP1*^*E4/IVS5*^ NSCs after the treatment with 20 μM δ-tocopherol. The images were taken with 40X objective lens. Data are displayed as mean ± SD. * *P* < 0.05, *** *P* < 0.001 compared to the untreated control

We further evaluated the effects of DT on cytoplasmic lipid droplet accumulation using the Nile red-staining assay measured with the yellow-gold fluorescence channel. We found that DT dose-dependently reduced the Nile red fluorescence signal in NCL patient NSCs. The effects on reduction of Nile red staining with the treatment of 20 uM δ-tocopherol ranged from 12.4% in the *TPP1*^*E4/E6*^ NSCs to 34.9% in the *TPP1*^*E4/IVS5*^ NSCs (Fig. 6a and b). The results indicated that DT significantly reduced the cytoplasmic lipid droplets accumulation in the NCL NSCs.

### Effects of ERT, DT and HPBCD on cellular levels of PPT1/TPP1 and subunit c

**Fig. 7:**
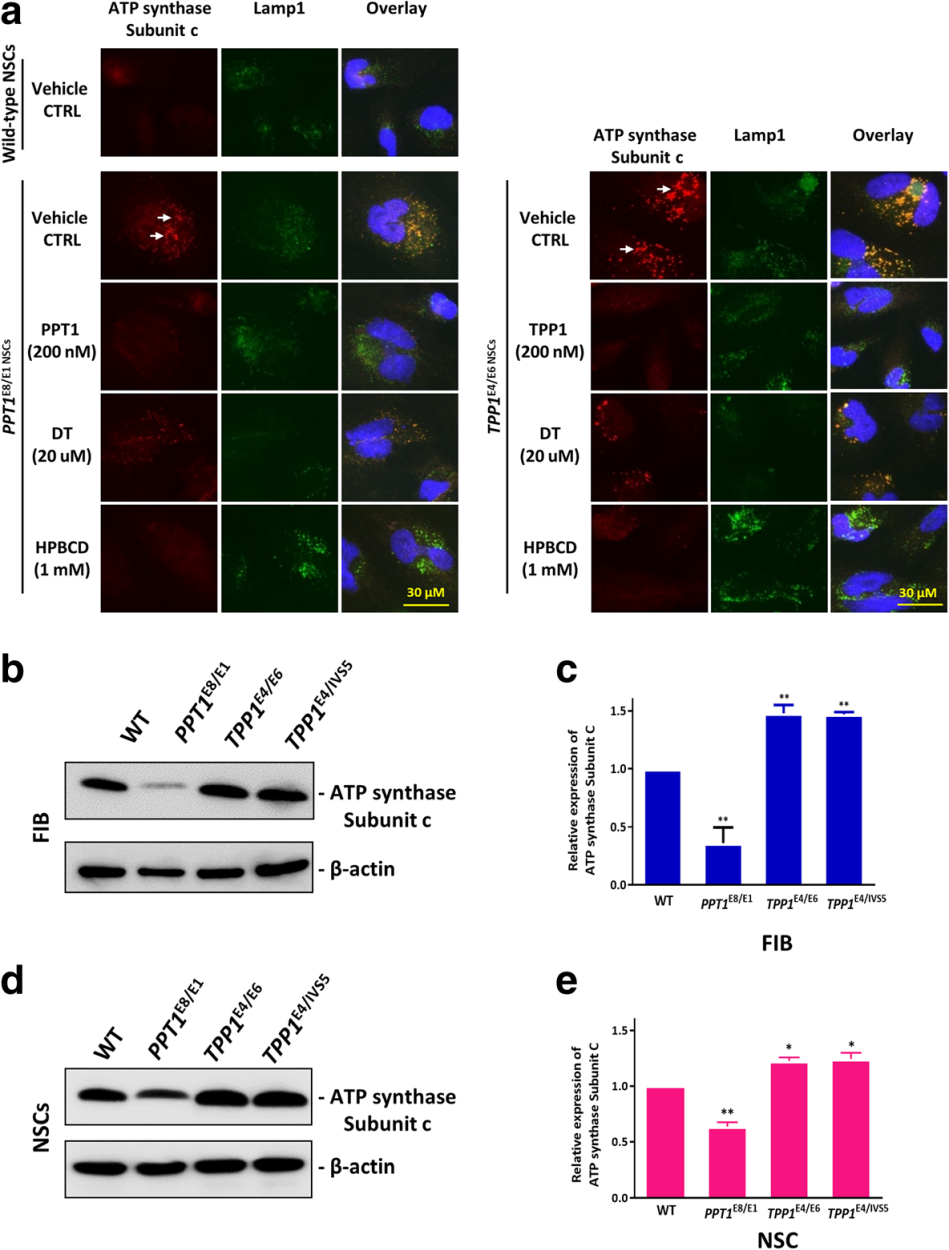
Co-localization of subunit c and Lamp1 in NCL NSCs and the expression of subunit c in NCL NSCs. **a** Co-localization of LAMP-1, a lysosomal marker, with subunit c in *PPT1*^E8/E1^, *TPP1*^E4/E6^, and wild-type NSCs. The cells were immunostained with antibodies recognizing subunit c (red fluorescence, see white arrows) and Lamp1 (green fluorescence). Minimal overlap of subunit c and Lamp1 immunostaining was observed in wild-type cells (yellow in overlay), but Lamp1 strongly, though not perfectly, overlaps with the accumulated subunit c in *PPT1*^E8/E1^ and *TPP1*^E4/E6^ NSCs. Treatment of INCL and LINCL NSCs with recombinant PPT1 and TPP1 decreased subunit c accumulation in lysosomes of patient cells, respectively. Similar effects were also observed in cells after treatments with δ-tocopherol and HPBCD. Blue represents Hoechst nuclei stain. Images were captured with 60X objective. **b** and **c** Expression of subunit c in NCL fibroblasts analyzed by the Western blot. The expressions of subunit c in *PPT1*^E8/E1^ fibroblast were weaker than WT, but the expressions of subunit c increased in *TPP1*^E4/E6^ and *TPP1*^E4/IVS5^ fibroblast compared to WT (**b**). It showed that subunit c expression was decreased by 64% in *PPT1*^E8/E1^ fibroblast, and increased 1.5-fold in both *TPP1*^E4/E6^ and *TPP1*^E4/IVS5^ fibroblast compared to WT (**c**). Data are the mean ± SEM. ** *P* < 0.01. Expression of subunit c in NCL NSCs (D and E). The expressions of subunit c were weaker in *PPT1*^E8/E1^ NSCs than WT, but the expressions of subunit c were increased in *TPP1*^E4/E6^and *TPP1*^E4/IVS5^ NSCs compared to WT (**d**). It showed that subunit c expression was decreased by 36% in *TPP1*^E4/E6^ NSCs, and increased 1.2-fold in both *TPP1*^E4/E6^ and *TPP1*^E4/IVS5^ NSCs compared to WT (**e**). Data are displayed as mean ± SD. * *P* < 0.05, ** *P* < 0.01, compared to the WT control

Abnormality in subunit c of mitochondrial ATP synthase and accumulation of subunit c were reported in the NCL patient cells although the pathogenesis of NCL has not been fully elucidated [4, 12, 21]. We found the subunit c positive puncta (accumulation of subunit c) in all patient NSCs (Fig. 7a). In both INCL and LINCL NSCs, immunostaining revealed that Lamp1 (a lysosome marker) was co-stained with subunit c and the puncta was enlarged, compared to the WT control. Moreover, the subunit c accumulation in INCL and LINCL NSCs decreased after treatment with PPT1 and TPP1 respectively. Similar effects were also observed after treatment with 20 μM of δ-tocopherol and 1 mM HPBCD (Fig. 7a). Furthermore, we found that the expressions of subunit c in *PPT1*^E8/E1^ fibroblast (Fig. 7b and c) and NSCs (Fig. 7d, e) were lower than that in the WT control. However, the expression of subunit c increased in the *TPP1*^E4/E6^and *TPP1*^E4/IVS5 E1^ fibroblasts (Fig. 7b and c) and NSCs (Fig. 7d, e) compared to that of WT control. The results indicated that the lysosomal accumulation of subunit c may be independent to the total amount of subunit c in patient cells.

**Fig. 8:**
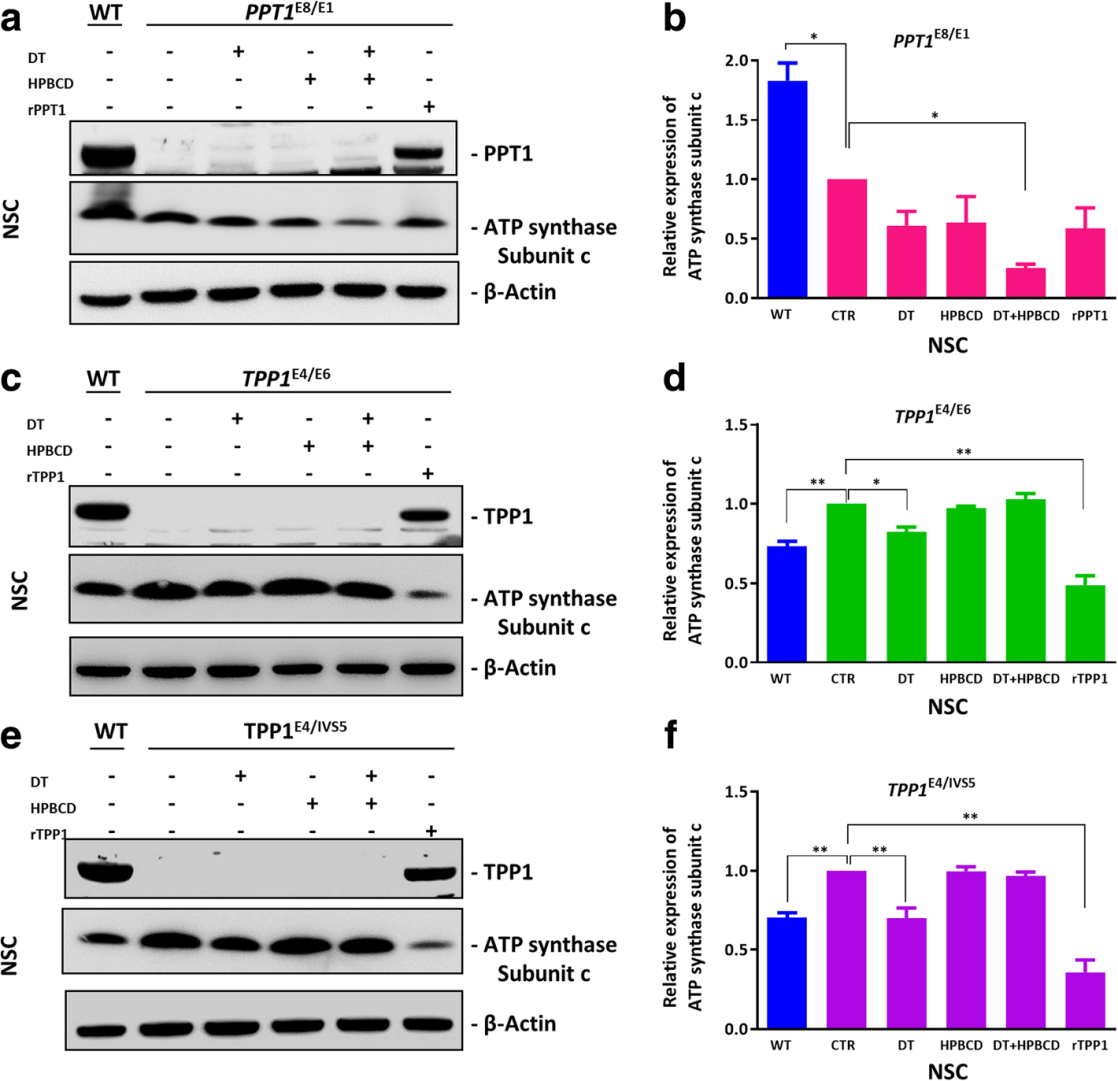
Effect of DT, HPBCD and enzyme replacement therapy on the accumulation of subunit c in patient NSCs. Cells were treated with 20 μM DT, 1 mM HPBCD, 20 μM DT plus 125 μM HPBCD, or 200 nM rPPT1/rTPP1 for 3 days. PPT1/TPP1 expression were restored after the PPT1/TPP1 replacement therapy. After the treatment with 20 μM DT plus 125 μM HPBCD, the expression of subunit c in *PPT1*^E8/E1^NSCs decreased by 75% (**a** and **b**). Moreover, it showed that subunit c expression in *TPP1*^E4/E6^ NSCs and *TPP1*^E4/IVS5^ NSCs was decreased by 51% and 64% respectively, in TPP1 replacement treatment, and also decreased by 18%, 30% with DT treatment (**c**, **d**, **e** and **f**). Data are displayed as mean ± SD. * *P* < 0.05, ** *P* < 0.01

After ERT with recombinant rPPT1 or rTPP1, the relevant enzyme level in these NCL NSCs significantly increased, confirming that the recombinant proteins entered the cells (Fig. 8a, c and e). However, δ-tocopherol and HPBCD didn’t alter the levels of PPT1/TPP1 in NCL NSCs, indicating that both compounds reduced enlarged lysosomes through different mechanism of action compared to ERT.

Western blot analysis revealed that when patient NSCs were treated, the subunit c protein level significantly decreased following treatment with ERT and a combination of δ-tocopherol and HPBCD in *PPT1*^E8/E1^ (Fig. 8a-b). ERT with rTPP1 protein decreased the subunit c level by 50% and 64% in the *TPP1*^*E4/E6*^NSCs and *TPP1*^*E4/IVS5*^NSCs, respectively. Subunit c levels decreased by 18% and 30% in the *TPP1*^*E4/E6*^ NSCs and *TPP1*^*E4/IVS5*^ NSCs, respectively (Fig. 8c, d, e, f), when treated with δ-tocopherol.

## Discussion

The neuronal ceroid lipofuscinoses (historically known as Batten Disease) are a group of inherited neurodegenerative disorders. Patient-derived neuronal cells are more biologically relevant model systems for study of disease pathogenesis and evaluation of compound efficacy for drug development. We have successfully generated iPSC lines from three NCL patient dermal fibroblast cell lines using the non-integrating CytoTune-Sendai virus reprogramming method. These iPS cells have been differentiated into NSCs which exhibited the characteristic NCL disease phenotype of, deficiency of the mutated enzyme, enlarged lysosomes and accumulation of subunit c in lysosomes. These iPSCs and NSCs exhibited normal morphology and growth rate compared to the WT control cells. Treatment of the patient cells with the relevant recombinant human enzyme can rescue the disease phenotype. The results demonstrate that these patient-derived NSCs can be used as a cell-based NCL disease model to evaluate drug efficacy.

Although ERT is currently available for the treatment of several lysosomal storage diseases, its effect is limited to the relief of peripheral symptoms as the recombinant proteins cannot penetrate into the central nervous system (CNS). Several delivery carriers have been reported that improved brain accumulation of recombinant enzymes [22]. However, these approaches for delivery of recombinant enzymes into the brain are still under early development and thus may not be employed for clinical treatment for several years. Brineura is the newly approved enzyme replacement therapy for TPP1 deficiency, CLN2 early this year. However, Brineura is administered to the cerebrospinal fluid (CSF) by infusion via a surgically implanted reservoir and catheter. It can only be administered by, or under the direction of a physician knowledgeable in intraventricular administration. Thus, it is inconvenient to patients and the treatment cost is very high.

Gene therapy has emerged as a potential treatment of lysosomal storage diseases. The viral vector can be injected intracranially into brain, resulting in the therapeutic effects of reducing lysosomal storage materials and rescuing cells from dysfunction [11]. The preclinical gene therapy study in the CLN2 defective mice revealed a restored normal level of TPP1 enzyme activity by CLN2-adeno-associated virus. The human trials have been initiated but it will take several years before conclusions can be drawn [23, 24]. Other therapeutic approaches are either ineffective, under development or with high risks including hematopoietic stem cell transplantation, substrate reduction therapy, immune therapy and pharmaceutical chaperone therapy [9, 25–28].

Application of patient iPSCs for modeling disease phenotypes has emerged as a new approach for drug discovery and development [29]. Many neurological diseases do not have disease relevant animal models which have greatly limited the use of compound screening and evaluation of drug efficacy for drug development. The recently available methods of differentiation of patient iPSCs to mature cells such as neuronal cells, cardiomyocytes and hepatocytes also provide new cell-based disease models for phenotypic drug screens [30]. To date, several human iPSCs were reported for 50 lysosomal storage diseases, including Gaucher [31], Mucopolysaccharidosis (MPS) type 1 [32], MPS IIIB [33], Pompe diseases [34], Fabry disease [35] and Niemann–Pick disease type C (NPC) [15]. The iPSC generation and neuronal differentiation has been recently reported for CLN2 and CLN3 [36], which examined the therapeutic effects of enzyme replacement therapy and a few small molecule in the patient derived cells. While the previously reported TPP1 activity inducers (Fenofibrate and gemfibrozil) did not increase TPP1activity, PTC124 (suppressing nonsense splicing mutations) increased the TPP1 activity and reduced the disease phenotype in patient iPSC-derived neural progenitor cells.

In this study, the differentiated NCL NSCs exhibited the characteristic disease phenotype of enlarged of lysosomal sizes, similar to the paired NCL patient derived fibroblasts. NSCs are self-renewable and can be produced in large quantities for compound screening. Compared with differentiated neurons, NSCs are more readily adapted into the high throughput screening for lead discovery and drug efficacy evaluation. Several laboratories have reported using iPSCs differentiated NSCs and neural progenitor cells for high throughput compound screening to identify lead compounds [37, 38]. Therefore, the patient derived NSCs are an appropriate disease model compared to the patient fibroblasts.

The results from ERT in this study demonstrate full efficacy of recombinant human enzymes on relief of disease phenotype in the patient derived NSCs. The data also demonstrated that CLN1 and CLN2 NSCs can be used as cell-based models for evaluating other drugs. Previous work shows that δ-tocopherol reduces lysosomal cholesterol accumulation in patient cells derived from NPC [13, 15]. The mechanism of action of the tocopherols was linked to an increase in lysosomal exocytosis in the patient cells [13]. Our present study revealed that δ-tocopherols dose-dependently reduced the enlarged lysosomes in both CLN1 and CLN2 NSC cells. HPBCD, a complex cyclic carbohydrate composed of seven sugar residues in a ring structure, was reported to reduce cholesterol accumulation in NPC cells [15, 39, 40]. The effect of HPBCD on NPC was also confirmed in animal models [41–43] and in clinical trials [44]. In the present study, the results showed that a high concentration of HPBCD was needed to alleviate the enlarged lysosomes in both CLN1 and CLN2 NSCs. The reported IC_50_ value of HPBCD in the NPC NSCs was in the low micromolar level which is a much lower concentration of HPBCD than the concentration required to reach IC_50_ (1 mM)in the CLN1 and CLN2 cells. We then tried the combination therapy of HPBCD with δ-tocopherol; this produced a synergistic effect on reduction of enlarged lysosomes in both CLN1 and CLN2 NSCs. The concentrations needed for each of the compounds in the combination were significantly reduced.

Protein sequencing of storage body proteins revealed a specific storage of subunit c from mitochondrial ATP synthase in lysosomes, first found in CLN6 [45], and extended to CLN2, CLN3, CLN5, CLN6, CLN7 and CLN8 [46]. In CLN1, the sphingolipid activator proteins (SAPs or saposins) A and D were reported as the major storage proteins in lysosomes, but not in subunit c, as revealed by gel electrophoresis and protein sequence analysis [5]. In this study, the protein level of subunit c decreased in CLN1 while it increased in the CLN2, as measured by Western blot analysis in both patient fibroblasts and NSCs. The lysosomal accumulation of subunit c was observed via immunohistochemical staining both in CLN1 and CLN2 NSCs. Treatment of patient cells with recombinant human enzymes restored the protein level of PPT1 or TPP1 in the patient NSCs, while reduced the level of subunit c. Treatment with δ-tocopherol, HPBCD, or a combination of HPBCD with δ-tocopherol did not increase PPT1 or TPP1 levels in patient cells. The amount of subunit c accumulation decreased by the treatment of δ-tocopherol or the combination of HPBCD and δ-tocopherol.

## Conclusions

Three lines of iPSCs have been generated from one CLN1 and two CLN2 patient dermal fibroblast lines. The neural stem cells derived from these patient iPSC lines exhibited characteristic disease phenotypes of deficiency of the relevant enzyme, enlarged lysosomes, lipid droplet accumulation and lysosomal storage of subunit c, which was rescued by the treatment with disease relevant recombinant enzymes and DT as well as being partially ameliorated by cyclodextrin HPBCD. These results demonstrate that the NCL patient iPSC derived NSCs are valid cell-based disease models with characteristic disease phenotypes that can be used for study of disease pathophysiology and drug development.

## Additional file

Additional file 1 **Figure S1.** Generation of Neuronal Ceroid Lipofuscinosis (NCL) induced pluripotent stem cells (iPSCs). **Figure S2.** Pluripotent stem cell protein markers analyzed by flow cytometry. **Figure S3.** Immunofluorescence staining of iPSC pluripotent stem cell protein markers. **Figure S4.** Protein marker expression in NSCs differentiated from WT control and NCL patient iPSCs. **Figure S5.** Enlarged lysosomes and lipid accumulation in NCL patient fibroblasts. **Figure S6.** Filipin staining in NCL fibroblasts and NSCs. **Figure S7.** Cytotoxicity of DT and HPBCD on NCL patient NSCs. **Figure S8.** Lysosomal pH indicated by a pHrodo™ pH sensor dye in NCL patient NSCs. (PDF 1842 kb)

## Abbreviations

ATP: Adenosine triphosphate
CNS: Central nervous system
CSF: Cerebrospinal fluid
DPBS: Dulbecco’s phosphate buffered saline
DT: δ-tocopherol
EDTA: Ethylenediaminetetraacetic acid
ERT: Enzyme replacement therapy
FACS: Fluorescence-Activated Cell Sorting
FIB: Fibroblasts
FITC: Fluorescein isothyocyanate
HPBCD: Hydroxypropyl-β-cyclodextrin
HRP: Horseradish peroxidase
INCL: Infantile neuronal ceroid lipofuscinoses
iPSCs: Induced pluripotent stem cells
LINCL: Late-infantile neuronal ceroid lipofuscinoses
MPS: Mucopolysaccharidosis
NCLs: Neuronal ceroid lipofuscinoses
NPA: Niemann-Pick type A
NPC: Niemann–Pick disease type C
NSC: Neural stem cell
PBS: Phosphate-buffered saline
PPT1: Palmitoyl-Protein Thioesterase 1
ROCK: Rho-associated coiled-coil kinase
rPPT1: recombinant WT PPT1
rTPP1: recombinant WT TPP1
TPP1: Tripeptidyl Peptidase 1
TR: Texas Red
WT: Wild type
