# Supplementary material for: Neural stem cells for disease modeling and evaluation of therapeutics for infantile (CLN1/PPT1) and late infantile (CLN2/TPP1) neuronal ceroid lipofuscinoses
Source: Orphanet J Rare Dis. 2018 Apr 10;13:54. doi: 10.1186/s13023-018-0798-2 (PMC5891977; doi:10.1186/s13023-018-0798-2)
Supplement: Supplementary file 1 — Figure S1. Generation of Neuronal Ceroid Lipofuscinosis (NCL) induced pluripotent stem cells (iPSCs). Figure S2. Pluripotent stem cell protein markers analyzed by flow cytometry. Figure S3. Immunofluorescence staining of iPSC pluripotent stem cell protein markers. Figure S4. Protein marker expression in NSCs differentiated from WT control and NCL patient iPSCs. Figure S5. Enlarged lysosomes and lipid accumulation in NCL patient fibroblasts. Figure S6. Filipin staining in NCL fibroblasts and NSCs. Figure S7. Cytotoxicity of DT and HPBCD on NCL patient NSCs. Figure S8. Lysosomal pH indicated by a pHrodo™ pH sensor dye in NCL patient NSCs. (PDF 1842 kb) [file 13023_2018_798_MOESM1_ESM.pdf]

Supporting Information for

Neural stem cells for disease modeling and evaluation of therapeutics for infantile (CLN1/PPT1) and late infantile (CLN2/TPP1) neuronal ceroid lipofuscinoses

Ni Sima[1,2], Rong Li[1], Wei Huang[1,2], Miao Xu[1], Jeanette Beers[3], Jizhong Zou[3], Steven Titus[1], Elizabeth A. Ottinger[1], Juan J. Marugan[1], Xing Xie[2], Wei Zheng[1†]

[1]National Center for Advancing Translational Sciences, National Institutes of Health, Bethesda, MD, USA. [2]Department of Gynecologic Oncology, Women's Reproductive Health Laboratory of Zhejiang Province, Women's Hospital, School of Medicine, Zhejiang University, Hangzhou, Zhejiang, P. R. China. [3]Center for Molecular Medicine, National Heart, Lung and Blood Institute, National Institutes of Health, Bethesda, MD, USA

†Corresponding author: wzheng@mail.nih.gov

Fig. S1

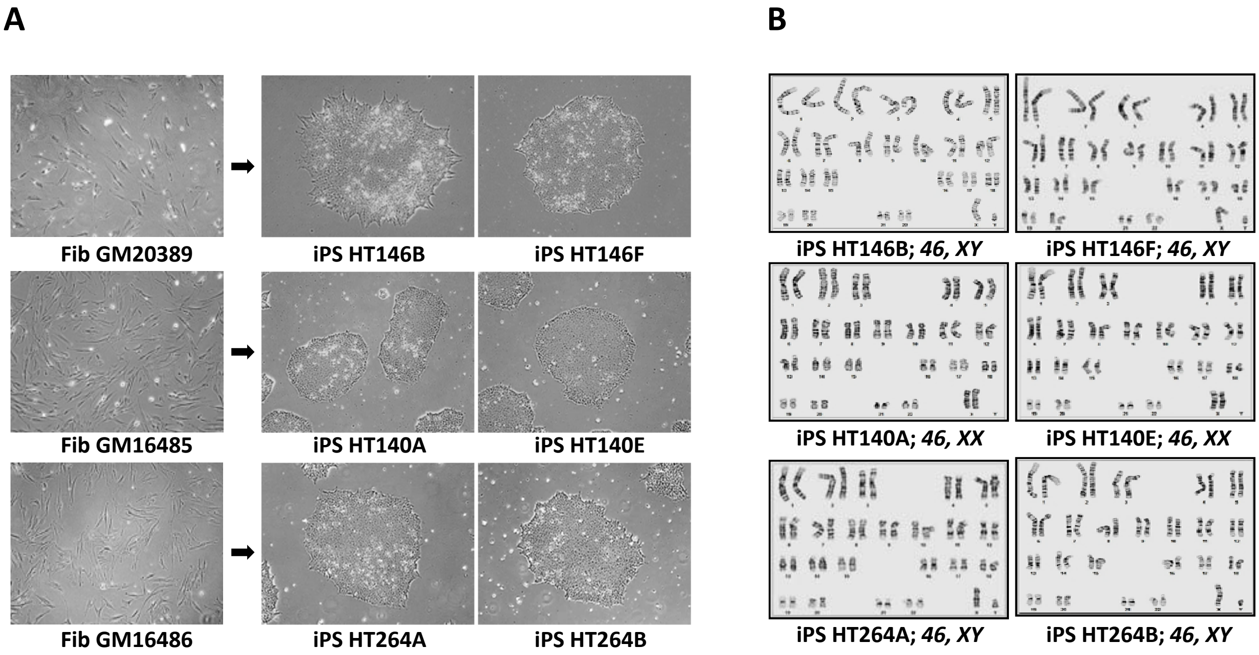

Supplementary Fig. 1. Generation of Neuronal Ceroid Lipofuscinosis (NCL) induced pluripotent stem cells (iPSCs). (A) Generation of NCL patient iPS cell clones from patient skin fibroblast cells. Phase contrast images of patient fibroblasts and iPS cells. (B) iPSCs derived from three NCL patient fibroblasts displayed normal karyotype.

Fig. S2

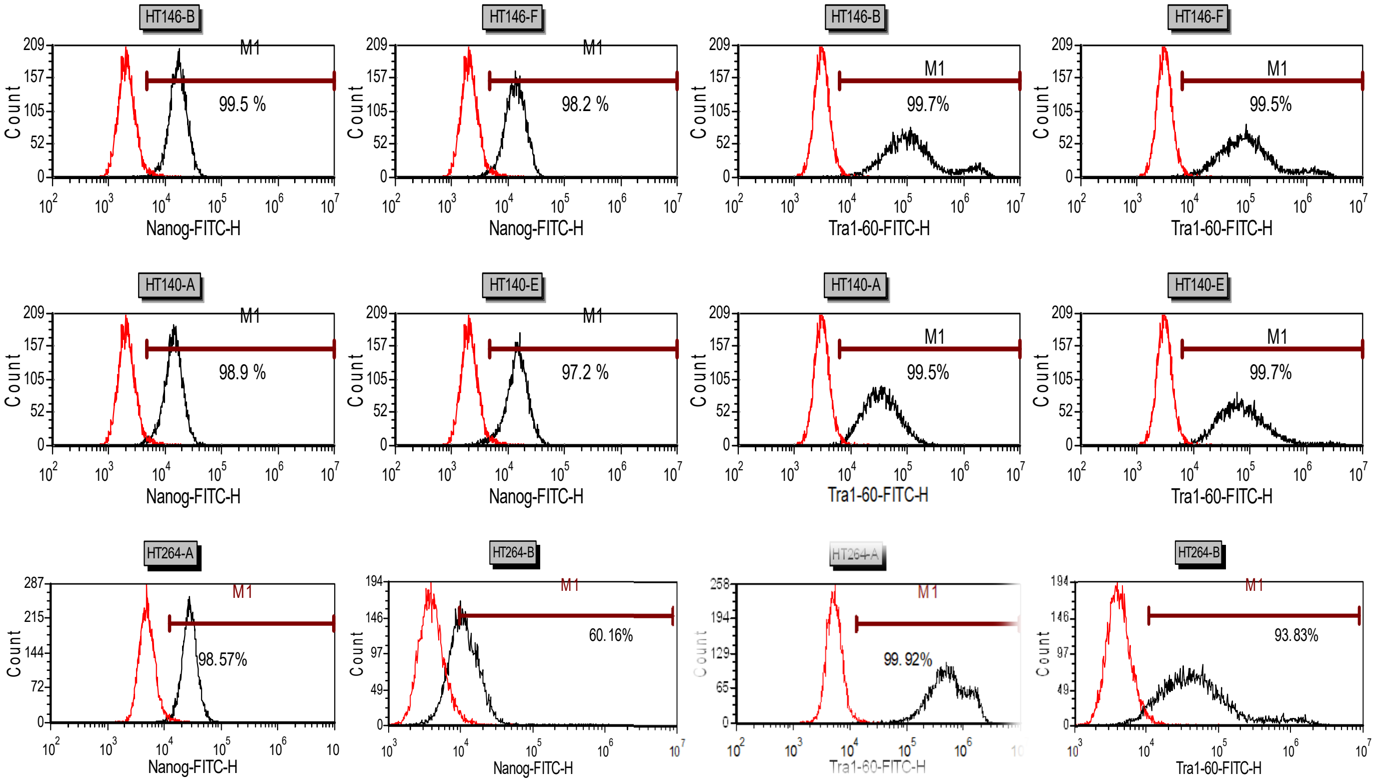

Supplementary Fig. 2. Pluripotent stem cell protein markers analyzed by flow cytometry. Cytometric analysis showed that these iPS cells expressed the pluripotency protein markers of Nanog and Tra-1-60.

Fig. S3

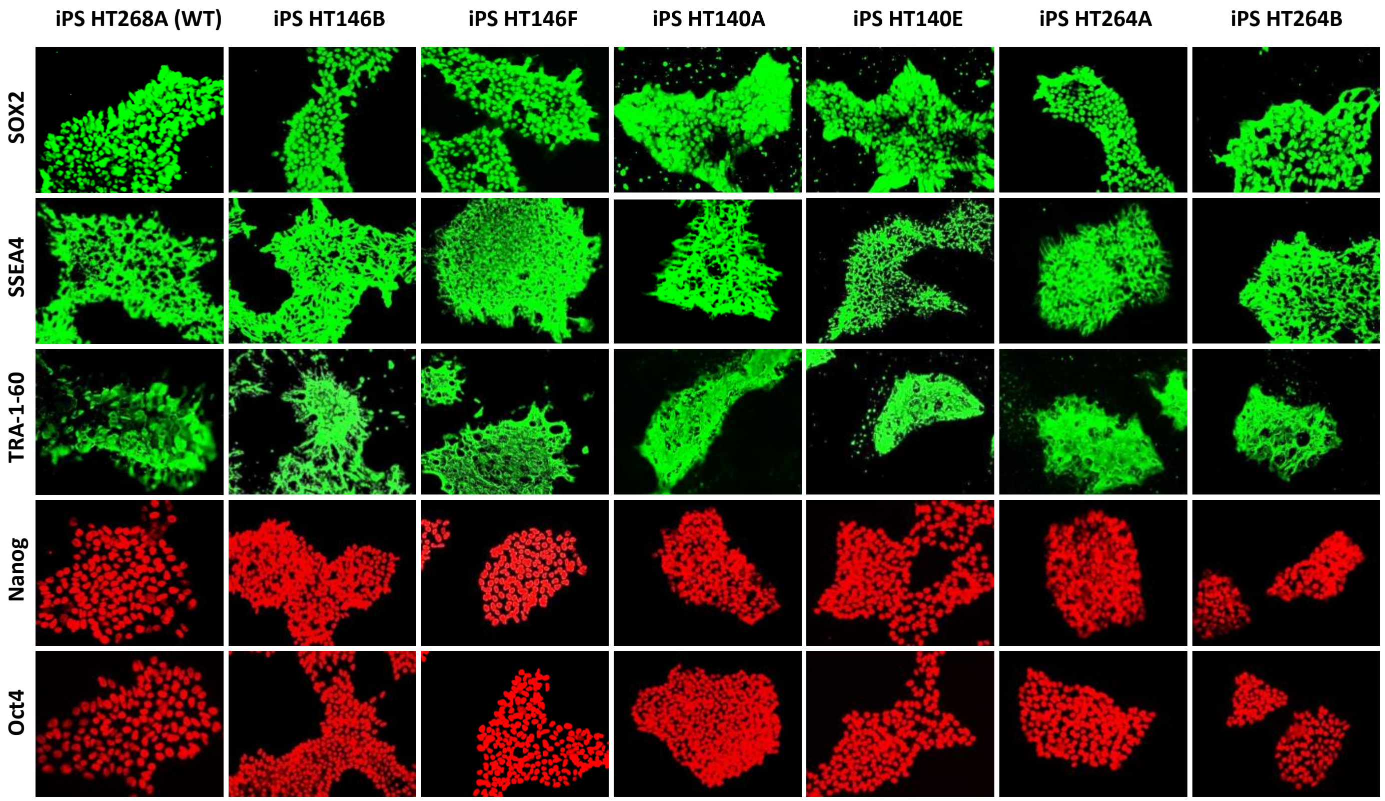

Supplementary Fig. 3. Immunofluorescence staining of iPSC pluripotent stem cell protein markers. The iPSCs derived from wild-type (WT) control and NCL patient fibroblasts were stained for the pluripotency markers of SOX2, SSEA4, TRA-1-60, Nanog and Oct4. The images were taken with 20X objective lens.

Fig. S4

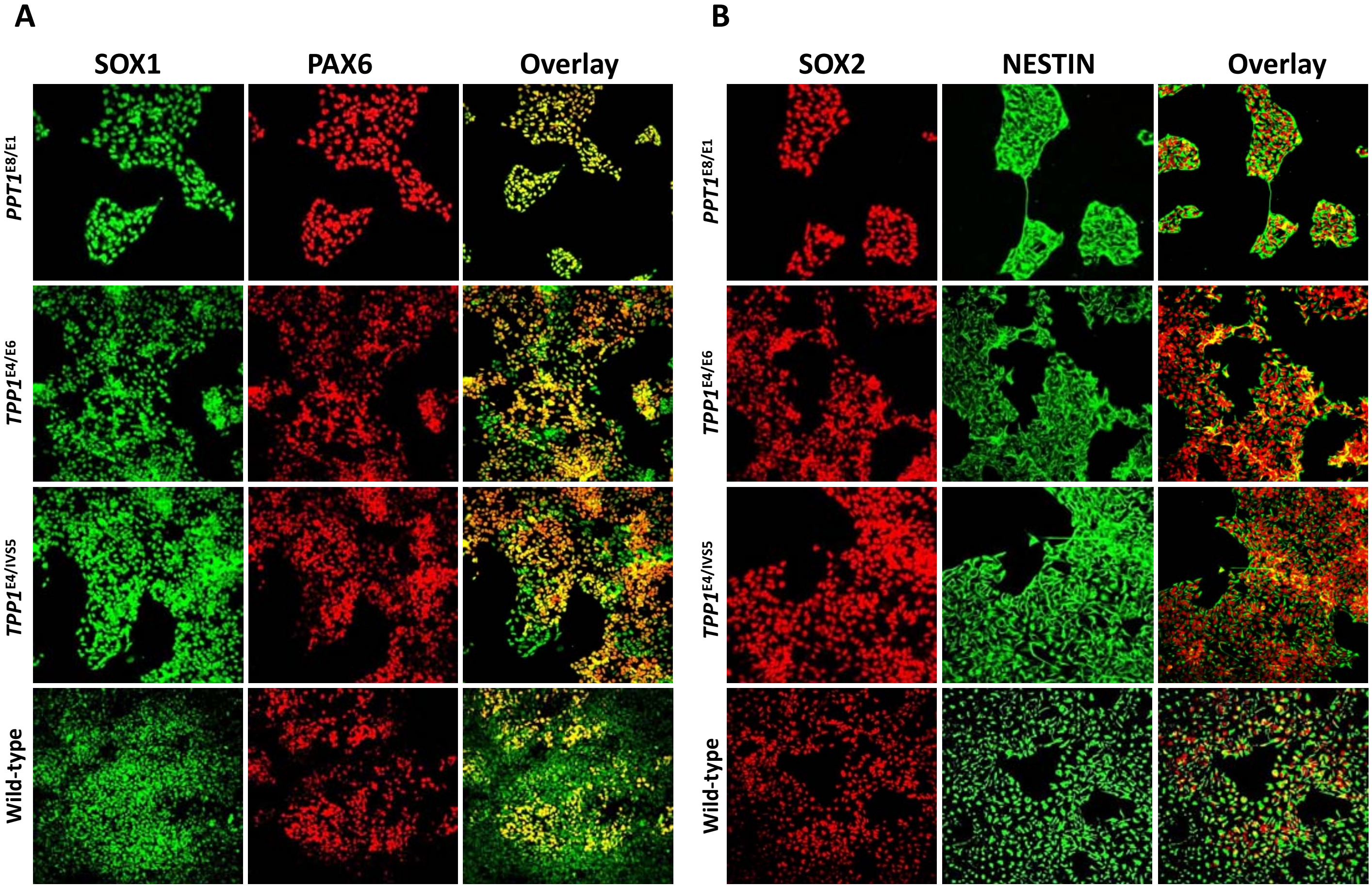

Supplementary Fig. 4. Protein marker expression in NSCs derived from WT control and NCL patient iPSCs. Representative images showed positivity for NSC marker proteins of Nestin, Sox1, Sox2 and PAX6. These NCL NSCs showed no morphological differences compared to WT control NSC cells. The images were taken with 20X objective lens.

Fig. S5

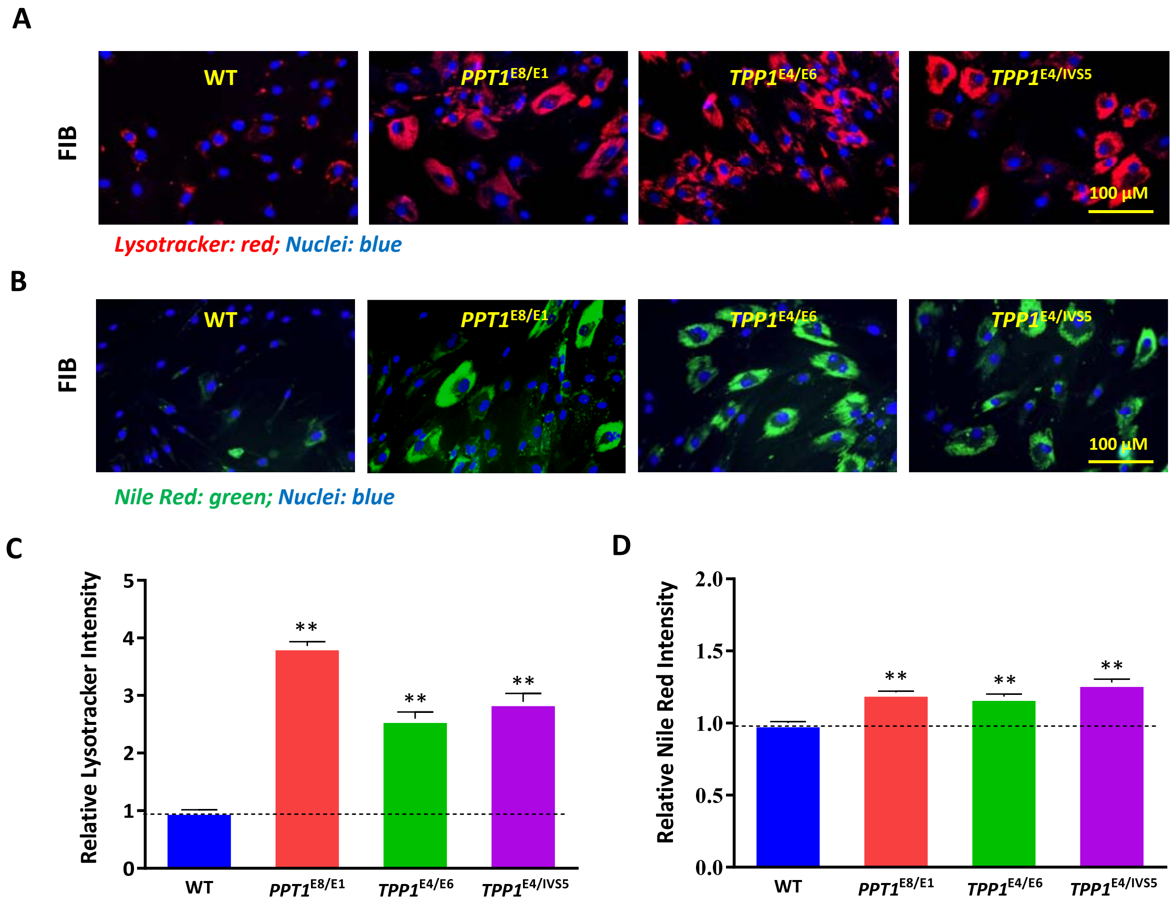

Supplementary Fig. 5. Enlarged lysosomes and lipid accumulation in NCL patient fibroblasts. Increased LysoTracker dye staining (A) indicates enlarged lysosomes and a strong Nile Red staining (B) indicates lipid accumulation. Enlarged lysosomes and lipid accumulation were observed in parental fibroblasts (FIB) of NCL patients compared to wild type (WT) control. Representative images of LysoTracker dye and Nile Red staining were shown with 20X objective lens. Quantification of the lysoTracker staining(C) and Nile Red staining (D) revealed significantly enlarged lysosomes and lipid accumulation in NCL patient fibroblasts. Data are displayed as mean $\pm$ SD. ** $P < 0.01$ vs. WT control.

Fig. S6

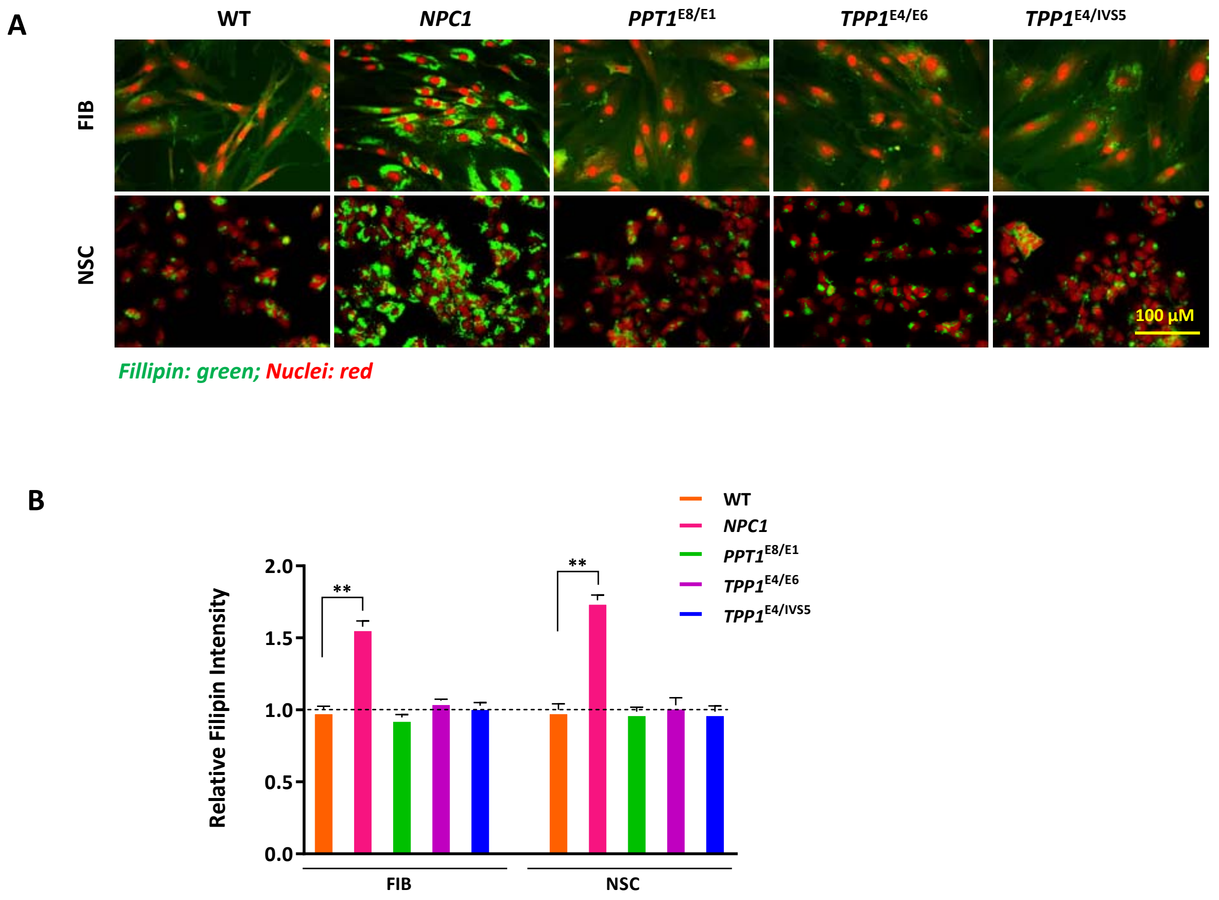

Supplementary Fig. 6. Filipin staining in NCL fibroblasts and NSCs. Representative images of Fillipin staining (A) showed no free cholesterol accumulation in patient NCL fibroblast and NSCs. A quantification of cholesterol accumulation in fibroblasts and NSCs was found no statistically significant (B). NPC1 patient fibroblasts and NSCs were used to serve as the positive control for Filipin staining. Data are displayed as mean ± SD. ** $P < 0.01$ vs. WT control.

Fig. S7

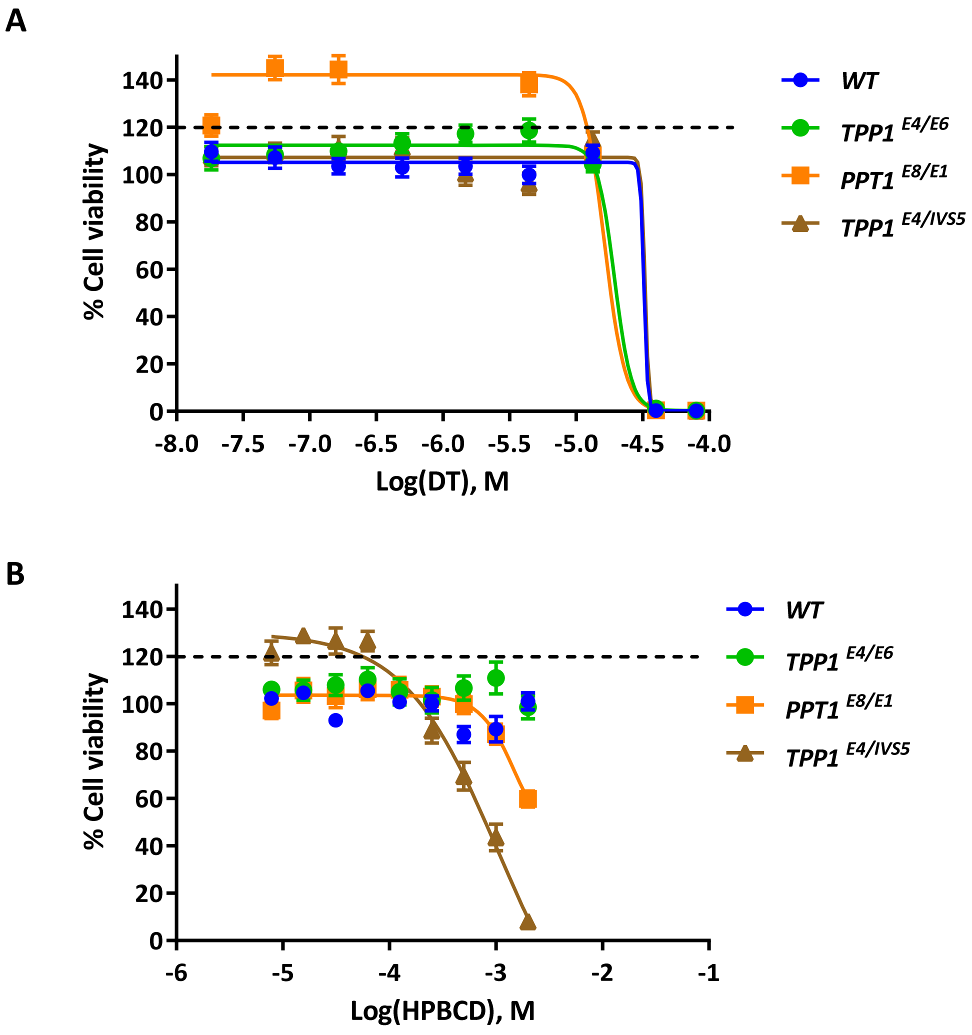

Supplementary Fig. 7. Cytotoxicity of δ-tocopherol (DT) and HPBCD on NCL patient NSCs. Cytotoxicity of DT (A) and HPBCD (B) was measured by an ATP content assay. DT lower than 20 uM concentration has no cytotoxic effect on all four NSCs cell lines. HPBCD showed no significant cytotoxic effect up to 1 mM concentration after a 3-day treatment period in those cells except for one of the NCL patient cell line, $TPP1^{E4/IVS5}$, which decreased the cell viability by 64.2%

Fig. S8

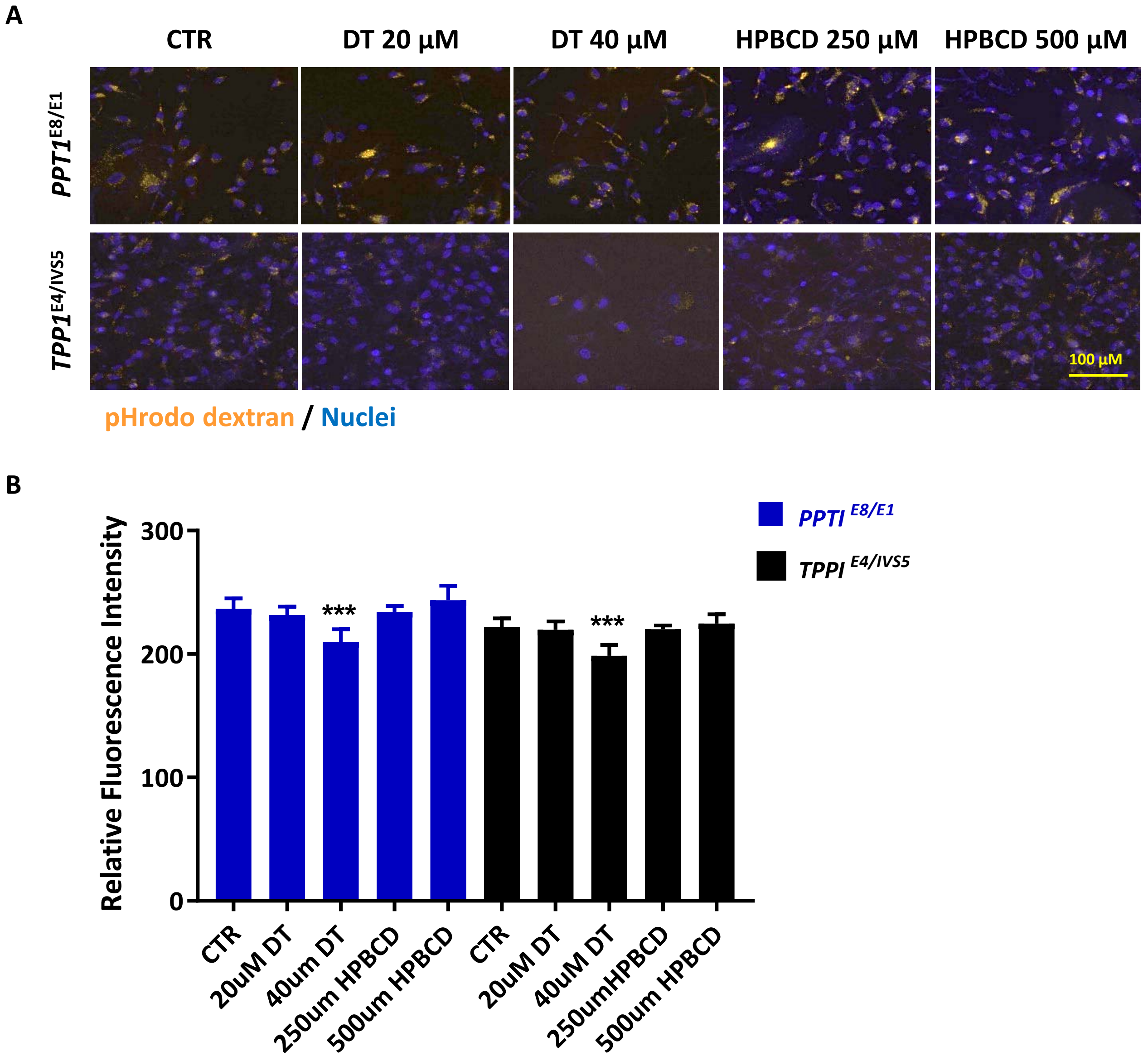

Supplementary Fig. 8. Lysosomal pH indicated by a pHrodo™ PH sensor dye in NCL patient NSCs. Representative images of pHrodo™ dextran staining (A) after 3-day treatment of DT and HPBCD. A quantification of pHrodo™ dextran fluorescence signal showed no significant lysosomal pH changes except for 40 µM of DT treatment, which showed small decrease of fluorescence intensity (B), possibly caused by the cytotoxic effect of high concentration of DT as indicated by the cell cytotoxicity assay. Data are displayed as mean ± SD. *** $P < 0.001$, compared to the untreated control.
